# Listeners and Readers Generalize Their Experience With Word Meanings Across Modalities

**DOI:** 10.1037/xlm0000532

**Published:** 2018-02-01

**Authors:** Rebecca A. Gilbert, Matthew H. Davis, M. Gareth Gaskell, Jennifer M. Rodd

**Affiliations:** 1 Department of Experimental Psychology, University College London, and MRC Cognition and Brain Sciences Unit, University of Cambridge; 2 MRC Cognition and Brain Sciences Unit, University of Cambridge; 3 Department of Psychology, University of York; 4 Department of Experimental Psychology, University College London

**Keywords:** language, lexical ambiguity, long-term priming, modality effects, semantic ambiguity

## Abstract

Research has shown that adults’ lexical-semantic representations are surprisingly malleable. For instance, the interpretation of ambiguous words (e.g., bark) is influenced by experience such that recently encountered meanings become more readily available (Rodd et al., 2016, 2013). However, the mechanism underlying this word-meaning priming effect remains unclear, and competing accounts make different predictions about the extent to which information about word meanings that is gained within one modality (e.g., speech) is transferred to the other modality (e.g., reading) to aid comprehension. In two Web-based experiments, ambiguous target words were primed with either written or spoken sentences that biased their interpretation toward a subordinate meaning, or were unprimed. About 20 min after the prime exposure, interpretation of these target words was tested by presenting them in either written or spoken form, using word association (Experiment 1, *N* = 78) and speeded semantic relatedness decisions (Experiment 2, *N* = 181). Both experiments replicated the auditory unimodal priming effect shown previously (Rodd et al., 2016, 2013) and revealed significant cross-modal priming: primed meanings were retrieved more frequently and swiftly across all primed conditions compared with the unprimed baseline. Furthermore, there were no reliable differences in priming levels between unimodal and cross-modal prime-test conditions. These results indicate that recent experience with ambiguous word meanings can bias the reader’s or listener’s later interpretation of these words in a modality-general way. We identify possible loci of this effect within the context of models of long-term priming and ambiguity resolution.

Lexical-semantic ambiguity is the rule rather than the exception: most words are ambiguous in that they can refer to different variations on a similar meaning (polysemes) or to completely different, semantically unrelated concepts (homonyms; Cruse, 1986; Lyons, 1977, 1981). For example, the polysemous word “run” has a cluster of related word senses such as those used in the phrases “the athlete/paint/politician/program runs,” whereas the word “bark” is a homonym with different unrelated meanings, as in the phrases “the bark of the dog/tree” (Klein & Murphy, 2002; Klepousniotou, 2002; Rodd, Gaskell, & Marslen-Wilson, 2002).

Skilled language comprehension therefore depends on the ability to disambiguate the precise meaning of individual words to build an accurate representation of the intended message. This is typically accomplished easily and fluently in adult native speakers, with rapid disambiguation occurring ‘on-the-fly’ during listening and reading. Although contextual cues are normally sufficient to indicate which meaning is correct, this disambiguation process is made easier by biases that promote preferential access to the most likely meaning (see Vitello & Rodd, 2015, for a recent review).

One of the strongest and most important biasing factors is the overall frequency with which the word is used to refer to each possible meaning in the language, that is, the relative meaning dominance. For instance, the word “pen” has one meaning (writing implement; dominant meaning) that is much more common relative to another (animal enclosure; subordinate meaning). Numerous studies have shown that interpretation of ambiguous words is biased toward the meaning that occurs most frequently. This bias is stronger when there is a greater imbalance in dominance, and it results in faster, less effortful access for high-frequency meanings (Vitello & Rodd, 2015). This increased availability of high-frequency meanings can be revealed most clearly in the absence of a biasing context, such as in simple word association tasks: participants are more likely to retrieve the more frequent meaning of an ambiguous word (e.g., produce the associate “write” rather than “pig” in response to “pen”; Twilley, Dixon, Taylor, & Clark, 1994).

Although the effects of immediate sentence context and meaning dominance on lexical-semantic disambiguation are well established (Twilley & Dixon, 2000; Vitello & Rodd, 2015), research suggests that lexical-semantic representations are more flexible and dynamic than previously thought. Not only are adults highly skilled at learning new meanings for previously unambiguous words (Rodd et al., 2013), but they are also able to update their representations of familiar word meanings based on their current linguistic experience. Specifically, recent experience with an ambiguous word in the context of one of its subordinate meanings has a strong influence on subsequent interpretation of the same word in the absence of biasing context, an effect referred to as *word-meaning priming* (Betts, Gilbert, Cai, Okedara, & Rodd, 2017; Rodd et al., 2016, 2013; see also Zeelenberg, Pecher, Shiffrin, & Raaijmakers, 2003 for a related finding). For instance, exposure to the sentence “The man accepted the *post* in the accountancy firm.” results in an increase in the probability of a later word association response to “post” that relates to the prime-consistent ‘employment’ meaning, compared with when the word meaning is not primed. This effect is strongest when the association test occurs within a few minutes of the presentation of the priming sentence (Rodd et al., 2016, Experiments 1 and 2), but it then becomes relatively stable and lasts for at least 20–40 min in lab-based experiments (Betts et al., 2017; Rodd et al., 2013, Experiment 1) and up to several hours in real world situations (Rodd et al., 2016, Experiments 1, 3, and 4).

Word-meaning priming relies critically on the presentation of the ambiguous word form itself during priming, rather than simply the semantic content related to the word’s meaning. The latter effect is semantic priming, which occurs over shorter timescales but does not produce an equivalent bias in word association responses after longer delays. Rodd et al. (2013, Experiment 3) found that replacing the ambiguous word with a synonym in the priming sentences (e.g., substituting a synonym for “post” as in “The man accepted the *job* in the accountancy firm”) produced a priming effect for job meanings of “post” after a 3 min prime-to-test delay, but not after a 20-min delay.

Previous studies have examined various possible explanations for word-meaning priming. For instance, the effect does not appear to be driven by a detailed exemplar/episodic memory for the priming sentences, given that there is no priming advantage when the encounters with the word form are presented using the same speaker compared with when two clearly different speakers (male vs. female) are used at prime and test (Rodd et al., 2016, Experiment 1; Rodd et al., 2013, Experiment 2). This finding is inconsistent with an account in which perceptual details are retained in the memory trace of the prime encounter and then matched to the test encounter, because such an account would predict that a same-voice test encounter will more strongly cue the memory of the prime (cf. an episodic lexicon account; Goldinger, 1996; Luce & Lyons, 1998).

In addition, word-meaning priming does not seem to be a direct consequence of the explicit recall of the prime sentences at test. If this were the case then one might expect a substantial number of word association responses to repeat words from the item’s prime encounter, but in fact these ‘repeated words’ are relatively rare and the priming effect remains after these responses are removed (Rodd et al., 2013, Experiments 1–3). Explicit recall of the priming sentences might be particularly likely when participants are aware of the priming manipulation, however previous studies show that priming effects were not dependent on participant self-reported awareness (Betts et al., 2017; Rodd et al., 2016). Furthermore, the salience of the ambiguity in the prime phase has not been shown to affect the presence or magnitude of the priming effect (Rodd et al., 2016, 2013).

These studies make clear that recent experience plays a key role in modulating the availability of word meanings by making low-frequency meanings more readily available after they have been recently encountered. This type of priming will facilitate communication in the likely scenario of word meanings being used consistently within conversations (Gale, Church, & Yarowsky, 1992). However, the underlying mechanism of word-meaning priming remains unclear. In the present study, we investigate which specific aspects of lexical-semantic representations are changed as a consequence of exposure to an ambiguous word in a particular meaning context. In what follows, we consider the possible mechanism(s) of word-meaning priming.

## Mechanism of Word-Meaning Priming

Word-meaning priming has two distinctive attributes that set it apart from other types of linguistic priming and thus constrain mechanistic explanations. First, it survives relatively long prime-test delays (at least 40 min; Rodd et al., 2016). By contrast, many types of linguistic priming can only survive very short prime-test intervals, ranging from no delay at all to only a few seconds and/or intervening items (e.g., semantic and form priming, but see Joordens & Becker, 1997, and Bowers, Damian, & Havelka, 2002, for possible exceptions). Short-term priming has been attributed to the influence of multiple, compound cues in short-term memory (STM) on retrieval from long-term memory (Ratcliff & McKoon, 1988), or residual transient activation of the prime stimulus representation within the lexical-semantic network (McClelland & Rumelhart, 1981; Morton, 1969). However, the compound-cue account requires the continued activation of all stimuli in STM between prime and test, which becomes unfeasible when priming effects are shown for a large number of items and with long prime-test delays. Similarly, a residual activation account predicts increased activation for representational units/nodes of all the stimuli encountered across the prime-test intervals, which would quickly result in indiscriminate, wide-spread activation across the whole network. For these reasons, the causal mechanisms involved in short- and long-term priming are thought to be distinct; the latter effect is often considered as a form of implicit learning (Becker, Moscovitch, Behrmann, & Joordens, 1997).

Longer-term priming effects can be simulated in computational models through small changes to connection strengths between lexical-semantic representational layers. In distributed connectionist models of word recognition (e.g., Hinton & Shallice, 1991), word forms and meanings are represented as sparse distributed patterns of activation, with connections between all word form and semantic units, as well as recurrent ‘clean up’ connections among the units within representational layers. When a word is presented to the network, activation flows from the input (word form) to the output (semantic representation) until the network settles into a stable representation (i.e., attractor state). Although persistence in the intrinsic activations of representational nodes/units can mimic short-term priming effects, changes to the connection strengths *between* them tend to produce smaller but lasting effects that are more resistant to interference from intervening stimuli (Becker et al., 1997). One way that these changes can take place is through error-correcting learning where connection weights are altered slightly to adjust for the difference between the network’s initial and target states (Becker et al., 1997; Rodd, Gaskell, & Marslen-Wilson, 2004). The result is that the network forms an ‘attractor state’ that makes the target state more likely to occur on the next encounter with the same input (word form). These alterations in the connection strengths among active units will affect the network’s response to words that are similar in form or meaning, while leaving the network’s response to other words unaffected.

A second distinctive feature of word-meaning priming is that it is not restricted to the same behavioral task at prime and test. Existing word-meaning priming studies have used semantic relatedness decisions to sentence-word pairs (Rodd et al., 2013) or passive listening (Rodd et al., 2016, Experiments 1 and 2) as the prime task, and word association as the test task. In addition, Rodd et al. (2016, Experiments 3 and 4) conducted naturalistic experiments where the prior exposures to ambiguous words and meanings occurred during a routine part of the participants’ lives (e.g., rowing-related meanings for individuals who participate in this sport), and thus there was no experimental priming task. The fact that word-meaning priming is not task-specific is important for ruling out a stimulus-response learning account of repetition priming, which could involve a different learning mechanism (Henson, Eckstein, Waszak, Frings, & Horner, 2014).

Under the assumption that the word-meaning priming effect is driven by long-term changes to connections within the lexical-semantic network, we can consider the specific locus of this effect. In what follows, we address this question within a distributed connectionist model of word recognition. We note, however, that similar distinctions could arise in any model that incorporates connections with variable weights, including models that use localist representations (e.g., the interactive two-step model, Dell, Schwartz, Martin, Saffran, & Gagnon, 1997).

## Locus of Word-Meaning Priming

**Figure 1 fig1:**
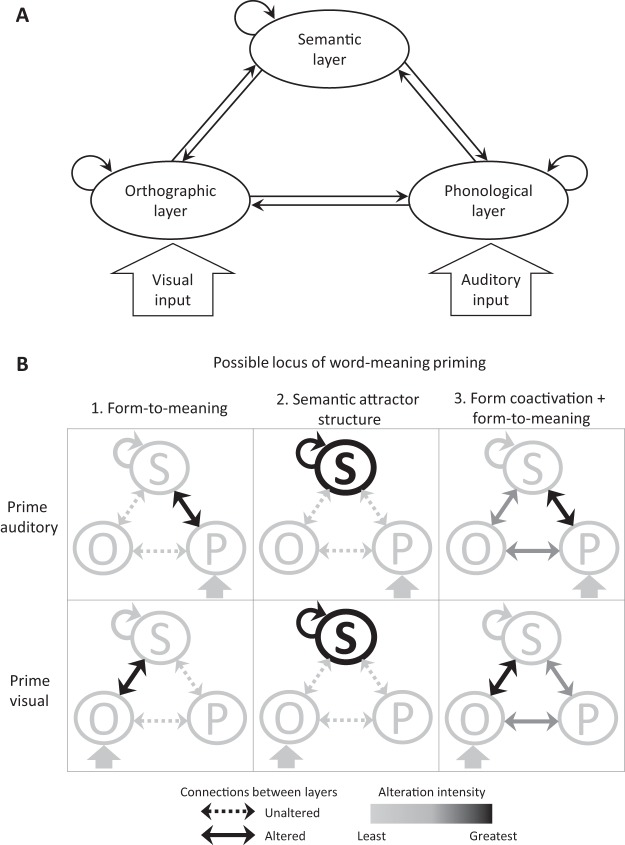
(A) Schematic representation of a triangle model of word recognition (Harm & Seidenberg, 2004; Seidenberg & McClelland, 1989). Circles represent distributed patterns of activation. Straight arrows represent the connections between layers, and circular arrows represent recurrent ‘clean up’ connections that form attractor states within the layer. (B) Columns show three possible loci of the word-meaning priming effect (see text). Each column depicts the changes within the system in response to auditory (top row) and visual (bottom row) prime encounters under each hypothesis.

We examine the potential locus of word-meaning priming within the context of a ‘triangle’ model structure, such as that used by many models of word reading and spoken word recognition (e.g., Seidenberg & McClelland, 1989). Many of these triangle models use the same distributed connectionist structure described in the previous section, where word forms and meanings are represented as sparse patterns of binary activation across a large set of units. The triangle structure results from connections between orthographic and phonological units (representing the written and spoken form of words), as well as connections between each of these layers and a shared lexical-semantic layer (see Figure 1A).[Fig-anchor fig1]

One particularly relevant implementation of such a model is that proposed by Rodd, Gaskell, and Marslen-Wilson (2004), which is capable of learning one-to-many mappings between single word forms and two meanings. Although Rodd and colleagues focused exclusively on the orthography-semantics mapping, here we discuss their proposed mechanism for learning multiple meanings of ambiguous words as it would occur within the full triangle model structure. The Rodd et al. (2004) network was trained through repeated exposures to all form and meaning units set to their target values [0 or 1], where on each exposure, an error-correcting algorithm was used to adjust the form-to-meaning connection weights, as well as the weights of recurrent connections between semantic units. After the network had been trained on all word-meaning patterns, a word form input pattern was presented to the network, with each semantic unit initially being set to [0], but then becoming activated on the basis of input from the active orthographic units. For word forms that had been paired with two meanings during training, the initial semantic activation was usually some combination of features from the word’s different meanings: a semantic ‘blend state.’ The model’s recurrent connections in the semantic layer prevented it from settling into these meaningless blend states by biasing the activation of semantic units toward those that commonly co-occurred with the currently active units during training (i.e., that were part of a single coherent meaning) and away from the units that rarely or never co-occurred. This resulted in stable attractor basins for each separate meaning pattern (e.g., a dog’s noise, the outer covering of a tree) that made the blend state unstable.

Within distributed connectionist models such as Rodd et al. (2004), alterations to connection strengths between units can potentially explain long-term priming effects. Even assuming this, there are still multiple possible loci of word-meaning priming. One explanation is that the form-to-meaning mapping is changed as a result of experience. Indeed, Rodd et al. (2013) suggested this possibility: “Within this [distributed connectionist] framework, any recent experience with one of the meanings would strengthen the connections between its form-based and semantic representations such that when the model next encounters the word’s form there is an increased probability of it settling into the recently encountered meaning” (p. 192, brackets added for clarity). By this account, the connections between the input form of the word (orthographic or phonological) and the semantic activation pattern are strengthened after the priming encounter so that a subsequent encounter with the same input pattern will be more likely to reactivate the most recently activated semantic pattern (i.e., the primed meaning). Others have also suggested that the form-to-meaning connections can account for similar types of long-term lexical-semantic priming (Monsell, 1985; Zeelenberg et al., 2003). A schematic representation of this possibility is shown in the first column in Figure 1B, with the predicted locus of change in response to an auditory (top row) or visual (bottom row) prime encounter with the ambiguous word.

A second possibility is that word-meaning priming results from a strengthening of the connections among semantic units (Figure 1B, second column). This is the explanation of long-term semantic priming proposed by Becker and colleagues (1997). The authors presented model simulations and empirical results supporting the view that experience with prime words deepens the attractor basins for those words in the semantic layer, and that the semantically similar target words are affected by this change because of their overlap in semantic space. In conjunction with their original form-to-meaning hypothesis, Rodd et al. (2016) also proposed this alternative account of word-meaning priming: “equivalent changes to the connections within the semantic layer could potentially make the attractor basin for that meaning more stable, relative to the alternative unprimed meaning.” (p. 34). In other words, the units that correspond to the semantic features of the more recently encountered meaning of an ambiguous word become more strongly connected to one another, forming a more stable attractor basin. This change to the attractor structure would make it more likely that, when the word is encountered again in the absence of any biasing context, the final settled state of the network would correspond to the primed meaning.

Critically, these two accounts make different predictions about whether word-meaning priming will transfer between auditory and visual modalities. The form-to-meaning connection hypothesis predicts that word-meaning priming will depend on the congruence of the word form (spoken or written) between prime and test encounters, with greater priming effects when the word form is the same modality. The strongest version of this account predicts that there will be no cross-modal priming, that is, when the ambiguous word is presented in different modalities in the prime and test exposures. The semantic attractor hypothesis, on the other hand, predicts that priming will impact on comprehension of word meanings regardless of the presentation modalities at prime and test, making it possible to observe cross-modal priming. Furthermore, if word-meaning priming is driven entirely by the connections between units in the semantic layer, then cross-modal priming should be equivalent to unimodal priming. Previous research on word-meaning priming (Betts et al., 2017; Rodd et al., 2016, 2013) has used only the spoken form at both prime and test, so at present there is no evidence for or against the existence of word-meaning priming in cross-modal conditions.

Thus far we have described two alternative loci of word-meaning priming within distributed connectionist models: changes to the weights of connections that (a) link word forms to meanings (i.e., between orthographic or phonological units and semantic units), or (b) form attractor states for meanings within the semantic layer. There is a third possibility, which is that the prime and/or test encounters with words in one modality (e.g., orthographic input in reading) involves the covert activation of the other modality (e.g., phonological recoding of written words, see third column in Figure 1B). There is substantial evidence for this type of word form coactivation from studies of silent reading and spoken word recognition (e.g., Chéreau, Gaskell, & Dumay, 2007; Newman & Connolly, 2004; Rastle, McCormick, Bayliss, & Davis, 2011). If this is the case, then it is possible for the form-to-meaning connections for *both* the phonological and orthographic representations to become strengthened as a result of a single encounter with the ambiguous word. Like the semantic attractor state hypothesis, this form coactivation account allows for the presence of cross-modal priming.

We note that these three accounts are not mutually exclusive. Rodd et al. (2016) suggested that changes to recurrent connections in the semantic layer could occur in conjunction with changes in the form-to-meaning mappings. Similarly, it may be the case that priming predominately affects the form-to-meaning connections within the presented modality, but that coactivation of the other word form also produces weaker changes to the form-to-meaning connections in the other modality—this possibility is depicted via the alteration intensity gradient in the third column of Figure 1B. Thus there are two important questions regarding the effect of prime and test modality on word-meaning priming; whether cross-modal priming can be observed, and if so, whether cross-modal priming is of equivalent magnitude to unimodal priming.

The prediction that unimodal and cross-modal priming will be equivalent is not specific to this interpretation within distributed connectionist models. This prediction could also arise from any model in which different word meanings are represented by localist, abstract ‘word-meaning nodes’ that are commonly activated by both auditory and visual input (Jastrzembski, 1981; Kellas, Ferraro, & Simpson, 1988). In these cases, word-meaning priming could result from increased availability of the primed word-meaning node (e.g., a raised threshold).

It is difficult to predict the effect of prime-test modality congruence on word-meaning priming because there have been relatively few investigations of modality effects in similar paradigms. Existing evidence suggests that long-term linguistic priming often involves both a modality-specific and modality-general component, but that the relative contribution of each varies across paradigms (Roediger & Blaxton, 1987; Rueckl & Galantucci, 2005). Some studies have shown weaker or absent long-term cross-modal repetition priming, specifically when performance on an auditory exposure task (e.g., word pleasantness ratings, sentence-word completion judgments, explicit memorization) and visual test task (e.g., lexical decision, word fragment/stem completion; McKone & Murphy, 2000; Monsell, 1985; Roediger & Blaxton, 1987; Rueckl & Galantucci, 2005) is compared with a visual unimodal condition.

There is also some evidence for an asymmetric pattern of modality effects in long-term priming. For instance, Monsell (1985) reported more cross-modal transfer in repetition priming from sentence completion judgments to lexical decision in the visual prime/auditory test condition compared with auditory prime/visual test. This was assumed to be the result of covert coactivation of phonological word forms during reading in the visual prime condition, coupled with little or no coactivation of orthographic word forms in the auditory prime condition. Although it may seem that the coactivation of phonological representations from orthographic forms should also occur during the visual test phase, resulting in equivalent priming for both cross-modal conditions, it is possible that orthographic-to-phonological activation is stronger when the task is unspeeded (as in the sentence completion priming task used by Monsell) or that it is task-dependent.

Further evidence of this cross-modal asymmetry comes from Rueckl and Mathew (1999), who showed that priming one heterographic homophone in a pair (e.g., week) within a disambiguated context increases the probability of responding with the other, unprimed word in a visual stem completion test task (e.g., ‘weak’ in response to wea__), where the test stem was always incompatible with the spelling of the primed homophone. This effect occurred regardless of the modality of the primed homophone, suggesting that covert activation of the (shared) phonological word form occurred during the prime and/or test phases. There was no significant priming in an orthographic similarity control condition (e.g., visually presented ‘teak’ as a prime for ‘weak’ in response to wea__), which suggested relatively little contribution of orthographic overlap between the homophone pairs in this priming effect. However, both the stem completion task and the lexical-decision task used by Monsell (1985) mainly depend on access to word forms, with relatively little dependence on semantics. The word-meaning priming tasks, on the other hand, require access to both word forms and their meanings. Hence, it remains unclear whether potential asymmetries in phonological/orthographic coactivation will be relevant to meaning access and selection from printed and spoken words.

The aim of the present study was to explore the locus of word meaning priming: does the change that occurs as a result of exposure to ambiguous word meanings reflect (a) a modality-specific change in form-to-meaning mappings, or (b) a modality-general change to lexical-semantic processing. We manipulate the modality of both the prime and test presentations within the word-meaning priming paradigm, using word association (Experiment 1) and speeded semantic relatedness decisions (Experiment 2) to test for preferred interpretations and the speed of meaning access for ambiguous words. In addition to providing specific information about the mechanisms of word-meaning priming, the results of this study will speak to more general issues concerning how readers/listeners use language experience to aid future comprehension. Is information about words and their meanings that is gained within one modality transferred in full to the other modality to (potentially) aid comprehension in that form of communication? Or do we accrue information about how words are used separately for speech and text?

## Experiment 1

In Experiment 1, we used the word association task as a test of meaning preference, as this allowed for a direct comparison with previous word-meaning priming experiments (Betts et al., 2017; Rodd et al., 2016, 2013). Following recent demonstrations that precise stimuli presentation timing and high quality reaction time (RT) data can be obtained when testing participants remotely using Web-based experimental platforms (Barnhoorn, Haasnoot, Bocanegra, & van Steenbergen, 2015; Hilbig, 2016; Pinet et al., 2016; Reimers & Stewart, 2015, 2016), the experiment was conducted using participants recruited and tested online.

There were two primary phases in the present experiment, separated by short filler tasks. In the prime phase, ambiguous words were presented within sentence contexts that supported a subordinate meaning (e.g., ‘The pig *pen* was muddier than ever’), and participants made semantic relatedness decisions to probe words presented after each sentence to ensure that they were attending to the sentences and processing their meanings. In the test phase, both primed and unprimed ambiguous words were presented in isolation, and for each word, the participant typed an associated word. By assessing which meaning of the ambiguous word these responses related to, we can determine how the word was interpreted during the test phase. Specifically, we were interested in whether the proportions of word association responses related to the primed meaning (e.g., associates to ‘pen’ that relate to the animal enclosure meaning) increased following presentation of an earlier priming sentence relative to when the ambiguous word had not been presented previously (unprimed). The sentences in the prime phase and single words in the test phase were presented in either auditory (spoken) or visual (written) form, and crossing these factors produced six prime-test conditions: auditory prime-auditory test, auditory-visual, visual-auditory, visual-visual, unprimed-auditory, unprimed-visual.

Consistent with previous word-meaning priming experiments that have used spoken materials at prime and test, we expected significant priming in the two unimodal conditions. Although the visual unimodal condition has only been previously examined in the context of cross-language priming (Poort, Warren, & Rodd, 2016), we have no reason to expect that the magnitude of unimodal visual priming will differ from that of the auditory unimodal condition. If word-meaning priming is driven entirely by changes in form-to-meaning connections (Figure 1B, first column), then we expect (a) a significant interaction between prime and test modality such that priming is reduced in the cross-modal compared with the unimodal conditions, and (b) no significant priming in the cross-modal conditions (auditory-visual vs. unprimed-visual, visual-auditory vs. unprimed-auditory). If word-meaning priming is primarily driven by changes to the semantic attractor structure (Figure 1B, second column) and/or to the form-to-meaning connections for both phonological and orthographic word forms (Figure 1B, third column), then we expect no significant difference between unimodal and cross-modal conditions (i.e., significant priming in the cross-modal conditions and no significant interaction between prime and test modality). Finally, if word-meaning priming involves a combination of modality-general and modality-specific components, then we would expect to see significant cross-modal priming combined with greater facilitation in unimodal conditions.

### Method

#### Participants

Eighty-one participants were recruited via Prolific Academic (Peer, Samat, Brandimarte, & Acquisti, 2015) and completed the experiment online. Participants indicated that they were native speakers of British English who were born and currently residing in the U.K. (verified with IP address geolocation). We used an *a priori* inclusion criteria of no less than 2 standard deviations below the mean percent correct in the semantic relatedness and vocabulary tasks, and a session duration of 60 min or less. This criterion resulted in the exclusion of three participants, who were replaced during data collection to reach the target of 13 participants per version (see Design). The final sample included 78 volunteers (39 women; *M* = 31 years, *SD* = 11, range = 18–56) who were paid £4.50 for their time. The study was approved by the UCL Department of Experimental Psychology Ethics Committee.

#### Design

We used a factorial crossing of three Prime Types (Auditory, Visual, Unprimed) and two Test Modalities (Auditory, Visual) which produced 6 Prime-Test conditions: Auditory-Auditory (AA), Auditory-Visual (AV), Visual-Auditory (VA), Visual-Visual (VV), Unprimed-Auditory (UA), Unprimed-Visual (UV). All participants were exposed to a subset of items in each of the 6 conditions, and all items were presented in all conditions across different versions of the experiment. This resulted in a crossed design which combined within-participant, between-item and within-item, between-participants manipulations. Participants were assigned to versions in counterbalanced order.

#### Materials

There were 78 ambiguous experimental items, which were a subset of the 88 items (words and priming sentences) used in Rodd et al. (2016) Experiment 2. Because each word was presented in both spoken and written form, only homonyms were used, that is, same pronunciation and spelling. This resulted in the exclusion of 10 items from the original stimuli list that had different spellings (e.g., night/knight). Meaning dominances were estimated as the proportions of unprimed word association responses relating to each meaning, based on a pretest conducted with a separate group of participants (*N* = 25). For each item, we selected one of the subordinate meanings (i.e., one that did not correspond to the highest proportion of the pretest word association responses) to be used in the priming sentence. The mean dominance of the primed meanings for experimental items was 0.23 (*SD* = 0.13, range = 0.00–0.48).

The 78 words were arranged in order of dominance and then pseudorandomly split into 6 lists of 13 words such that all lists contained words with similar dominance distributions (see Table 1 in the Appendix). Across the 6 versions, each word list was assigned to 1 of the 6 experimental conditions (see Table 3 in the Appendix). The dominance ratings did not differ across the 6 lists, *F*(5, 72) = 0.01, *p* = .999.

Each experimental item was presented in a sentence that used the item in a subordinate meaning context. The ambiguous words were disambiguated by their prior context to facilitate their comprehension, for example, “The musician had altered the song’s *key* several times.” Each sentence was paired with a written probe word, which for half of the sentences was related to the meaning of the sentence and for the other half of sentences was unrelated (see Table 4 in the Appendix). The sentences had a mean length of 9.3 words (*SD* = 1.7, min = 6, max = 13). The mean duration of the spoken sentences was 2.21 seconds (*SD* = 0.38, min = 1.40, max = 2.99).

The sentence and single word audio files used were the same as those used in Rodd et al. (2016), which were recorded in a sound-attenuated booth by a female native speaker of southern British English. All audio files were matched for RMS amplitude.

#### Procedure

**Figure 2 fig2:**
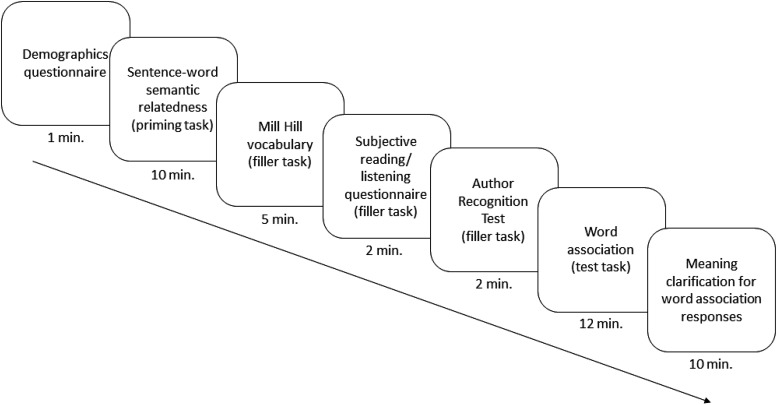
Experiment 1 order and approximate duration of tasks.

The experimental tasks were presented using an online survey tool (Qualtrics, www.qualtrics.com) and a JavaScript engine for collecting RTs (Barnhoorn et al., 2015). After an initial consent and demographic eligibility screening, participants completed the following tasks, which are shown in Figure 2 and explained in more detail below: semantic relatedness prime, vocabulary test, subjective reading/listening questionnaire, Author Recognition Test (ART), word-association test, meaning clarification. The whole session lasted 45 min on average. The vocabulary test, subjective reading/listening questionnaire and ART resulted in a delay of ∼9 min between the end of the prime task and beginning of the word association task. The mean estimated delay between the prime and test encounters (i.e., the mean difference between the midpoints of the two tasks) was 19.5 min.[Fig-anchor fig2]

##### Demographic questionnaire

Participants were asked to provide their age, gender, native language, country of birth and country of residence. The experiment ended at this stage if the participant did not meet the eligibility criteria.

##### Semantic relatedness priming task

Participants both heard (Auditory Prime) and read (Visual Prime) sentences containing an ambiguous word within the same randomized blocks of trials. After each sentence, a probe word appeared and the participant made a semantic relatedness judgment. For instance, the participant might read/hear the sentence “The pig pen was muddier than ever” and then read the probe word “ANIMALS.” On Auditory presentation trials, a fixation cross appeared in the middle of the screen while the sentence audio played. In the Visual trials, the sentence was written in the middle of the screen in sentence case (i.e., with the initial letter of the first word capitalized). After each sentence, participants moved on to the probe word by pressing the ‘f’ key. Presentation was self-paced to allow participants sufficient time to ensure that they understood the sentence before moving on to the probe word (this was particularly important for the Visual trials due to individual differences in reading speed). However, it was not possible to end the audio sentence before the entire file had played, or to move on from the visual sentence within the first 1000 ms of its onset. After the ‘f’ key press there was a 500-ms fixation cross, followed by the presentation of the probe word (in UPPER CASE) in the middle of the screen along with reminders for the response keys (‘j’ = related, ‘k’ = unrelated) below the probe word. Participants made a key press to indicate whether or not the probe word was related to the sentence. This task was designed to be relatively easy and the probes were never related to the inappropriate meaning of the ambiguous word.

This task began with a set of 16 practice sentences, all containing filler ambiguous words. Accuracy feedback was given after each response to the probe, and a warning message was shown if the response took longer than 2 seconds. The main task was made up of 56 sentences containing experimental items in the 4 priming conditions (AA, AV, VA, VV). The stimuli were separated into two blocks within the priming and test tasks, and the items were always presented within the same block for both tasks to reduce the between-item variance in prime-to-test duration. Roughly half (6 or 7) of the 13 target words within conditions were assigned to either block 1 or 2, resulting in similar numbers of trials per condition in the two blocks. The items within each condition that were assigned to block 1 or 2 in versions 1, 2, and 3 were assigned to the opposite block in versions 4, 5, and 6. Within blocks, the order of trials was uniquely randomized for each participant.

At the end of this task, we assessed participants’ awareness of the ambiguous words and the priming manipulation. Participants were asked via open-ended questions whether they (a) noticed anything in particular about the sentences they just heard or (b) had any ideas as to what the experiment was about. Participants were allowed to move on without responding.

##### Vocabulary test

This task was included so that we could exclude any participants who were not proficient speakers of English and who may therefore not have known all the subordinate meanings of the words in the experiment. The original version of the Mill-Hill vocabulary test (Raven, Raven, & Court, 1998) contains 34 target words with 6 words presented as multiple choice options, and the participant is asked to select the synonym from the set of 6 options. Three target words and seven incorrect multiple choice options overlapped with the experimental word set, either in form or meaning, so these words were removed (targets) or replaced with other words matched for length and frequency (incorrect options). The final version contained 31 trials.

##### Subjective reading/listening experience

This task was included with the aim of assessing the extent to which any cross-modal priming might be mediated by reading style (see Results). Participants responded to 12 statements using a 1–5 scale: 1 = *never or almost never*, 2 = *occasionally*, 3 = *sometimes*, 4 = *usually*, 5 = *always or almost always*.

##### Author recognition test

This task was included with the aim of assessing the extent to which any cross-modal priming might be mediated by reading experience (see Results). A modified version of the ART (Acheson, Wellu, & MacDonald, 2008) was used to assess the participants’ exposure to print. This test consists of 130 names, 65 of which are real authors and 65 are foils. Participants tick a box next to any name that they know to belong to a real author. This test was modified following the recommendations of Acheson et al. (2008) to include more popular authors, and to adapt the ART for British participants. Fifteen authors from the recently updated list (Moore & Gordon, 2015) who were identified as having the least discriminability were replaced with authors selected from the 2010–2013 British best-seller lists.

##### Word-association test

Participants were presented with written and spoken single words, and for each word, their task was to type an associated word into a text box. The instructions provided some example responses for an unambiguous probe word and encouraged participants to respond quickly during the task. The task was split into two blocks, with trials randomized within blocks. Items were presented in the same block as in the priming task. The two lists of unprimed experimental items (UA, UV) were split evenly across the two word association blocks and presented randomly with the other trials.

In the auditory test conditions, an audio player appeared in the middle of the screen and the sound played automatically. Participants were able to replay the word using the audio controls. On visual trials, the target word appeared in written form in the middle of the screen.

After this task, we again assessed participants’ awareness of the priming manipulation. Participants were asked whether they had any ideas as to what the experiment was about. As with the earlier awareness questions, this was an open-ended question and participants could move on without giving any response.

##### Meaning clarification

This task was included to facilitate the coding of word association responses. In each trial, a written experimental item appeared with the participant’s response, and a multiple choice question clarified the meaning of the item as it relates to their response. For example, if a participant entered the word ‘tree’ in response to the item ‘bark’ in the word association task, the participant would see: “You heard the word BARK, and your response was TREE. Which meaning were you referring to?” The multiple choice options were short definitions related to the dominant meaning (e.g., dog noise), the subordinate meaning (e.g., outer covering of tree), and an ‘other meaning’ option. There was also a “misheard the word” option for auditory test conditions only. The order of dominant/subordinate meaning definitions were randomly allocated to the 1st and 2nd options, whereas ‘other’ and ‘misheard’ were always the 3rd and 4th options, respectively. The order of trials was randomized for each participant.

#### Response recoding

The self-coding of responses was checked by Rebecca A. Gilbert and another researcher, both of whom are native speakers of English and were blind to condition. The first coder examined the word association responses and verified that the response categorization given in the meaning clarification task (dominant, subordinate, other meaning) was correct. In cases where the coders could not verify the intended meaning (e.g., ‘train’ in response to ‘coach,’ which might refer to either meaning), the participant’s self-coding was always used. Responses where the participant misheard the word or responded with the same word as the target were coded as a 4th category, ‘invalid.’ The first coder flagged the meaning clarification responses that were considered to be errors, for example, if the participant’s word association response seemed to unambiguously relate to one meaning, and where this was not consistent with the self-coding response. These cases were then examined by the second coder, and the two coders reached consensus.^1^

Of the total 5850 word association responses (78 participants × 75 items; see Main Analysis), the codes for 282 responses (4.8%) were changed from the participants’ self-coded values. Of these recoded responses, most (84%) were changes from a self-coding of ‘other meaning’ to one of the two definitions, or vice versa. A minority (14%) were recoded because they clearly related to one definition but were self-coded as the other definition. The remaining recoded responses (2%) were rare cases in which the participant reported mishearing the word but the response was clearly related to one of the definitions, or in which the response was invalid (e.g., the participant clearly misheard the word, responded with the target word).

### Results

Proportions of correct responses in the semantic relatedness priming task were high (*M* = 0.97, *SD* = 0.03, range = 0.85–1.00) showing that participants were attending to and successfully comprehending the priming sentences. Proportions of correct responses on the vocabulary test were in the normal range (*M* = 0.60, *SD* = 0.13, range = 0.23–0.97). We will not describe the results for the two remaining filler tasks (subjective reading/listening questionnaire, ART). These tasks were included to determine whether the hypothesized unimodal priming advantage was modulated by individual differences. As there was no significant increase in priming for unimodal versus cross-modal conditions (see Main Analysis), we did not investigate individual differences in the interaction.

#### Main analysis: Word association data

Three items were removed from the analysis because they were mistakenly included, despite having different levels of ambiguity in the two modalities: (‘break’ which can also be spelled ‘brake,’ ‘bow’ which can be pronounced to rhyme with ‘now’ or ‘know,’ ‘record’ which can be pronounced with first or second syllable stress). Responses that were coded as ‘invalid’ (1.5**%** of the data) were removed, which left 5763 out of 5850 (78 participants × 75 words) word association responses in the analysis.

Each response was coded as ‘1’ if its meaning was consistent with the meaning used in the priming sentences and ‘0’ if it related to any other meaning (i.e., either ‘dominant’ or ‘other meaning’). The average proportion of subordinate responses for the unprimed conditions was 0.23.^2^

**Figure 3 fig3:**
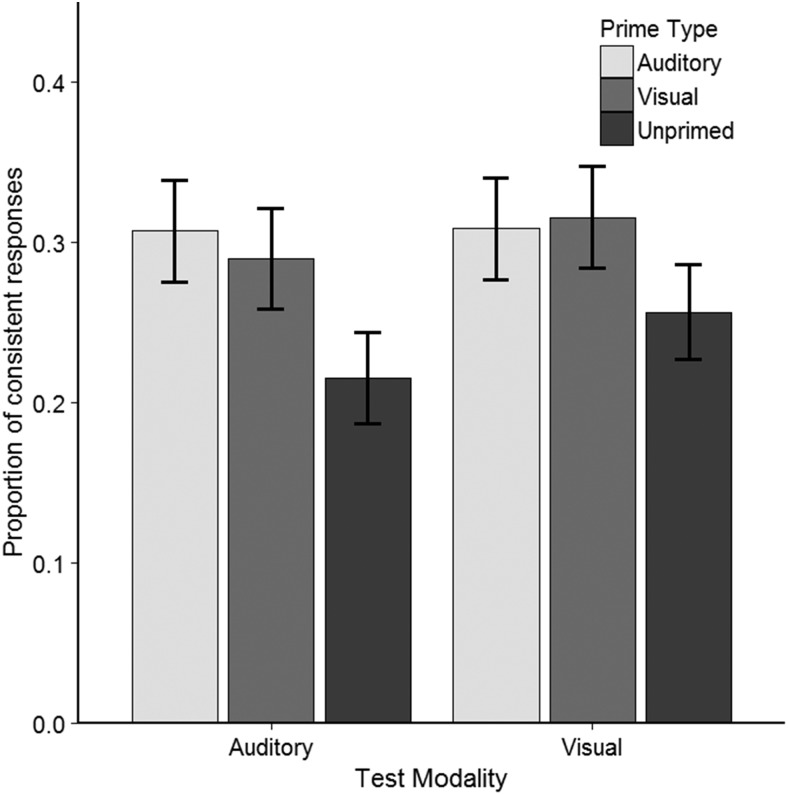
Experiment 1 proportions of word-association test responses that were consistent with the primed meanings across the 6 conditions. The Auditory (left bars) and Visual (right bars) Test Modalities are grouped on the *x* axis. Prime Type is color-coded: Auditory Prime (light gray), Visual Prime (medium gray), and Unprimed (dark gray). Bars show the subject grand means, and error bars show 95% CIs, adjusted to remove between-subjects variance (Morey, 2008).

Mean proportions of sentence-consistent responses within the 6 Prime Type × Test Modality conditions are shown in Figure 3. The responses were analyzed with a logistic mixed effects (LME) model using the ‘lme4′ package (Bates, Mächler, Bolker, & Walker, 2015) and R statistical software (version 3.3.1; R Core Team, 2013). The model took a 3 × 2 design with fixed effects for Prime Type (Auditory, Visual or Unprimed), Test Modality (Auditory or Visual), and the interaction. Within Prime Type, Helmert coding was used to define two planned contrasts: (a) Unprimed = −2/3 versus Auditory or Visual = 1/3, and (b) Auditory = −1/2 versus Visual = 1/2. Deviation coding was used for Test Modality: Auditory = −1/2 versus Visual = 1/2.[Fig-anchor fig3]

Our general approach to the LME modeling for both Experiment 1 and 2 was as follows. We first attempted to use the maximal random effects structure, given that the inclusion of all random slopes for fixed effects reduces the probability of Type I errors (Barr, Levy, Scheepers, & Tily, 2013). If this model did not converge, we removed the correlations among random effects terms, followed by the by-item and by-subject random intercepts. If these models did not converge, we removed the random effects term that accounted for the least variance, continuing in this manner until the model converged. All tests of fixed effects were then evaluated using models with this random effects structure. This method for dealing with nonconvergence is similar to the data-driven approaches proposed by Bates, Kliegl, Vasishth, and Baayen (2015) and Matuschek, Kliegl, Vasishth, Baayen, and Bates (2017) in that it reduces model complexity by removing terms that account for the least variance. However, in our case we stopped model reduction as soon as the model converged, whereas Bates et al. and Matuschek et al. sought to find the optimal model complexity given the data (even if it is not the most complex model that will converge).

In the present experiment, the final model contained a by-subject and by-item random intercept.^3^ Likelihood ratio tests were used to evaluate main effects and interactions, and significance of individual model coefficients were obtained using the *z* statistic in the model summary. The main effect of Test Modality was significant, β = 0.14, *SE* = 0.07, *z* = 2.20, *p* = .028, χ^2^(1) = 4.78, *p* = .029, though the difference was small (an increase of 0.03 in the model-adjusted mean proportions for visual over auditory test). The main effect of Prime Type was significant, χ^2^(2) = 36.90, *p* < .001. The coefficient for the first Prime Type contrast (Unprimed vs. the two primed levels) was significant, β = 0.42, *SE* = 0.07, *z* = 6.02, *p* < .001, and the coefficient for the second Prime Type contrast (Auditory Prime vs. Visual Prime) was not significant, β = −0.03, *SE* = 0.08, *z* = −0.40, *p* = .692. Bonferroni-corrected Wald *z* tests for pairwise comparisons within Prime Type were conducted using the ‘glht’ function from the ‘multcomp’ package for R (version 1.4–6; Hothorn, Bretz, & Westfall, 2008). The results of these tests were consistent with those from the model summary: both the Auditory Prime (*z* = 5.48, *p* < .001) and Visual Prime (*z* = 5.09, *p* < .001) conditions were associated with significantly more sentence-consistent responses than the Unprimed conditions, and the Auditory and Visual Prime conditions were not significantly different from one another (*z* = −0.40, *p* > .999).

A likelihood ratio test for the full fixed effects model and a model without the interaction term showed that the critical Prime Type × Test Modality interaction was not significant, χ^2^(2) = 2.65, *p* = .265. The significance tests for model coefficients showed that Test Modality did not significantly interact with either the first Prime Type contrast (i.e., unprimed vs. primed, β = −0.19, *SE* = 0.14, *z* = −1.36, *p* = .173), or the second Prime Type contrast (i.e., Auditory Prime vs. Visual Prime, β = 0.14, *SE* = 0.15, *z* = 0.91, *p* = .366). One limitation of null-hypothesis significance testing is that nonsignificant results are not interpretable, because a lack of sufficient evidence for rejecting the null hypothesis does not constitute evidence for the absence of an effect. For this reason, we compared the relative unstandardized effect sizes for the priming effect (i.e., first Prime Type contrast, unprimed vs. primed) and the critical interaction between the second Prime Type contrast (i.e., Auditory Prime vs. Visual Prime) and Test Modality. The model coefficient and 95% confidence interval for the priming effect was 0.42 [0.29, 0.56], while for the prime-test modality interaction this was 0.14 [−0.16, 0.44], which represents ∼33% of the size of the priming coefficient. We also used a Bayesian analysis to follow up this null interaction result, as this allowed us to quantify the likelihoods of each hypothesis against the other, given the data. Bayes Factors were computed using the Bayesian Information Criterion (BIC) approximation from two competing LME models.^4^ The alternative (1) model contained the full fixed effects structure, and the null (0) model lacked the 2 × 2 interaction between the Prime Type second contrast (A vs. V) and Test Modality (A vs. V). To compute the Bayes Factor in favor of the null, we used the formula *BF*_01_ = *e*^ΔBIC^_10_^/2^, where ΔBIC_10_ is the BIC for the alternative model minus BIC for the null model (Masson, 2011; Wagenmakers, 2007). This analysis showed that, given the data, the null hypothesis (that there is no Prime Modality × Test Modality interaction) was about 49 times more likely than the alternative hypothesis, *BF*_01_ = 49.40.

To determine whether there was significant priming in the two cross-modal conditions (AV and VA), we compared the proportions of sentence-consistent responses in each of these conditions to that in the unprimed condition with the same test modality (UV and UA, respectively). For each subset analysis, a full model was constructed with condition (cross-modal primed or unprimed) as a deviation-coded fixed factor and with by-subject and by-item random intercepts and slopes for condition. The full model did not converge for the Auditory Test Modality subset, so the by-subject intercept was removed as it accounted for the least variance. The model coefficient significance tests and model comparisons showed that there were significantly more consistent-meaning responses in both cross-modal conditions relative to the unprimed conditions with the same test modality (AV vs. UV: β = 0.34, *SE* = 0.15, *z* = 2.37, *p* = .018, χ^2^(1) = 5.30, *p* = .021; VA vs. UA: β = 0.51, *SE* = 0.15, *z* = 3.48, *p* < .001, χ^2^(1) = 10.62, *p* = .001).

We had not predicted a difference in the unprimed conditions as a function of test modality, however there appeared to be a numerical difference between the UA and UV subject means. In addition, the model coefficient for Test Modality was significant, and the pattern of means suggests that this effect may have been driven by differences in the Unprimed conditions. We therefore conducted an exploratory analysis to determine whether this difference was reliable. Using the ‘phia’ package for R (version 0.2–1; Rosario-Martinez, 2015), we tested of the effect of Test Modality within each level of Prime Modality, where the estimates and variances for each Prime Type × Test Modality condition were calculated from the full LME model. This analysis showed that there were significantly more sentence-consistent (subordinate) word association responses in the UV condition compared with the UA condition, χ^2^(1) = 5.28, *p* = .022. Although this difference was not expected, it does not affect the interpretation of the modality congruence effects of interest. We therefore defer further consideration of this difference until the General Discussion.

#### Participant awareness

In response to the awareness questions after the priming task, none of the participants predicted the priming manipulation and 2 participants expressed an awareness of the ambiguity. After the word-association test, 10 participants mentioned ambiguity, 8 participants indicated an awareness of the priming (e.g., repetition of words within the experiment, influence of the earlier task on later task performance), and 4 participants mentioned both ambiguity and priming.

To determine whether the results were driven by the ‘aware’ participants, the main analysis was repeated with only the ‘unaware’ participants. Participants were scored as ‘aware’ if they mentioned the priming manipulation and/or presence of ambiguity in response to any of the awareness questions. This categorization was intended to exclude either type of awareness to obtain a more conservative ‘unaware’ group. There were 23 (30%) aware and 55 (70%) unaware participants. The results of this subset analysis were consistent with the main analysis and similar analyses in previous studies (Betts et al., 2017; Rodd et al., 2016): the ‘unaware’ participants showed significant priming effects and no significant interactions between prime and test modalities (see supplemental materials for a detailed description of these results).

### Discussion

We examined the effects of prime and test presentation modalities on word-meaning priming, using proportions of word association responses that are consistent with the primed (subordinate) meaning as a measure of word-meaning preference. We found significant effects of priming, with more prime-consistent word association responses for words presented in primed than unprimed conditions. These results replicate those of previous auditory unimodal priming experiments (Betts et al., 2017; Rodd et al., 2016, 2013) and show for the first time that cross-modal word-meaning priming can be observed. This is also the first time that the word-meaning priming paradigm has been used in a fully Web based experiment. The effect of priming on sentence-consistent word association responses observed here (a raw increase of 6.9% from unprimed to primed in the subject means) is similar to that from a previous lab-based experiment with a comparable design (7.8% subject mean increase in Rodd et al., 2016; Experiment 2, 20- and 40-min delay conditions), suggesting that the switch to the Web based method did not result in a substantial reduction in the observed priming effect.

There was no significant interaction between prime and test modalities, meaning that there was no reliable priming advantage for unimodal over cross-modal conditions. Because null effects are difficult to interpret within a null hypothesis significance testing framework, we also computed a Bayes Factor, which showed that the data were more consistent with the absence of an interaction. In addition, separate analyses comparing each cross-modal condition to the unprimed condition with the same test modality showed significant priming in both cross-modal conditions. Taken together, these results provide initial support for the view that recent experience with ambiguous words biases the subsequent interpretation of these words in a modality-general way. Thus, the results of this experiment are incompatible with a strong version of the form-to-meaning account of word-meaning priming (Rodd et al., 2013, 2016), which predicts greater priming for unimodal over cross-modal prime-test conditions.

The word association task used in Experiment 1 is a measure of preferences for ambiguous words’ meanings. This is useful because it provides a measure of the individual’s word interpretation in the absence of any biasing context. However, there are a few disadvantages of the word-association test. One is that each trial produces a single binary response (either the associate is related to the primed meaning or not), and this discrete categorization may conceal subtle changes in word-meaning preferences that are insufficient to change interpretation from the default (dominant) meaning to the primed meaning. Consequently, this task may lack the sensitivity necessary to observe modality congruence effects, which could account for our null finding. A second issue is that the word association task is unspeeded; participants are free to reflect on their response and change it before committing. Thus it is possible that shifts in meaning preferences that are observed using the word association task are, in part, the result of strategic responding, rather than online access to word meanings. A third issue is that word association responses may be contaminated by association types that are not purely semantic, such as collocations/phrasemes and idioms (e.g., “rock and roll,” “armed to the teeth”), and priming could potentially reflect changes in the availability of these other types of associates. In Experiment 2 we use a different test task and sought to replicate the results of Experiment 1 using a speeded measure of word-meaning access.

## Experiment 2

Experiment 2 used a test task in which participants made speeded semantic relatedness judgments about individual ambiguous words and subordinate-related probe words (Cai et al., 2017). Compared with word association, this task provides a more sensitive, graded measure of the speed and ease with which readers can access specific word meanings for ambiguous words. In this task, the target ambiguous word is presented in spoken or written form, followed by a written probe word related to the primed (subordinate) meaning, for example, toast—SPEECH. Participants must indicate via one of two key presses, as quickly and accurately as possible, whether or not the meanings of the two words are related. It thereby provides a more ‘online’ measure of interpretation compared with word association responses.

One challenge associated with using this speeded relatedness task was that the primed words could potentially benefit from form-based priming compared with the unprimed words. Repetition priming experiments show that word forms are more quickly recognized if they have been recently seen/heard. In the present experiment, having previously encountered an auditory or visual word form earlier in the study may increase the recognition speed of that same word form in the test trials. If this is the case, then it could result in a modality-specific benefit to semantic relatedness decisions, if the relatedness decision to the second word (the probe) is influenced by the recognition speed of the first word (the target). Critically, this potential benefit for primed words would not reflect word-meaning priming. However, we expected form-based priming to have a negligible effect on responses during the semantic relatedness test task because repetition priming studies using lexical decision show reduced or absent facilitation effects when the word is initially presented in a sentence context (Levy & Kirsner, 1989; MacLeod, 1989; but see also Monsell, 1985) and when a homograph is presented in the context of different meanings at prime and test (Masson & Freedman, 1990). Furthermore, in the present experiment, speeded responses are made to a subsequently presented probe word, not to the ambiguous target word (which is the word that would benefit from form-based priming). Thus, even if initial processing of the target is facilitated due to form-based priming, the task still requires subsequent processing of the probe word and access to semantics to make a relatedness decision.

A second challenge for this task was equating stimulus timings in the auditory and visual test modalities. The time-course of target word recognition differs for spoken and written words, so the relative timing of the probe word onset will also differ between the two presentation modalities even when using the same stimulus onset asynchronies (SOAs). Studies using similar methods have shown that response patterns can vary as a function of the SOA (e.g., Henderson, Snowling, & Clarke, 2013). This effect of SOA is thought to reflect the time-course of activation and selection of ambiguous word meanings, with an initial activation of all meanings followed by the selection of one meaning and inhibition of others. To reduce the possibility of observing large differences between test modalities, the SOAs were roughly matched in the auditory and visual test conditions. However, a main effect of test modality would not be surprising, nor would it be interpretable because of differences in the timing of spoken and word recognition. Importantly, our main research question relates to the *interaction* between prime and test modalities, which should still be observable in the presence of a main effect of test modality.

Based on the results of Experiment 1, we expect that the probability of interpreting the ambiguous words with their sentence-consistent meanings will increase after priming, resulting in more accurate ‘related’ responses for the primed targets and subordinate-meaning probes at test. If priming also increases the speed of access to the primed word meanings then we expect faster correct responses for primed versus unprimed target words. Furthermore, if word-meaning priming affects modality-general aspects of lexical-semantic representations then, consistent with the results of Experiment 1, we should observe statistically equivalent priming in unimodal and cross-modal conditions. In contrast, if our failure to observe a significant prime-test modality interaction in Experiment 1 was a consequence of the limitations of the word association method (e.g., lack of sensitivity, influence of off-line/strategic processes), then here we might see this interaction due to faster and/or more accurate responses for unimodal over cross-modal conditions.

### Method

#### Participants

The recruitment methods, exclusion criteria, and payment were the same as in Experiment 1. For this experiment we also set an inclusion criteria of response accuracy within 2 standard deviations of the mean percent correct in the test task. Based on an unpublished pilot experiment with a similar test task but without the priming manipulation, we set an *a priori* sample size of 30 participants per version (180 in total). Seven participants were excluded and replaced during data collection (4 for exceeding the time limit, 1 for a low vocabulary score, 1 for low test task accuracy, and 1 for low prime and test task accuracies), and there was 1 extra participant as a result of accidental overrecruitment. The final sample included 181 volunteers (87 women; *M* = 29.5 years, *SD* = 8.3, range = 18–49). The study was approved by the UCL Department of Experimental Psychology Ethics Committee.

#### Design

The design was the same as Experiment 1, except that additional fillers were added to the semantic relatedness prime and test tasks (see Materials).

#### Materials

The items and sentence materials were the same as those from Experiment 1, with some exceptions described here. Five items from Experiment 1 were replaced with new items (see Table 1 and Table 2 in the Appendix). The semantic relatedness test required the addition of filler items (words and sentences) for both the priming and test tasks. This was because all experimental items were ‘yes’ targets in the test task, so an equal number of ‘no’ filler targets were needed that did not differ from the ‘yes’ targets in their overall proportions of item ambiguity or familiarity (via priming). Thus there were an additional 78 ambiguous filler items (52 primed, 26 unprimed) and 24 low-ambiguity filler items (all primed) added to this experiment (see Table 5 in the Appendix). The low-ambiguity filler items were included to reduce the salience of ambiguous words. The filler trials contained equal numbers of prime and test modalities, which were the same across all versions of the experiment. Responses to these additional fillers were not analyzed.

The new semantic relatedness test task required written probe words for all target items. For experimental items and half of the low-ambiguity fillers, the probe word was related to the primed meaning, and all others were unrelated (see Table 6 in the Appendix). The related probes were selected from common word association responses and dictionary definitions. These words were not used in the item’s priming sentence or as the relatedness probe following the priming sentence, nor were they related to the item’s other meaning(s). The related and unrelated probes were matched for number of letters (related: *M* = 5.60, *SD* = 1.72, unrelated: *M* = 5.59, *SD* = 1.81, *t*(178) = −0.04, *p* = .966) and log frequency per million (related: *M* = 3.72, *SD* = 0.69, unrelated: *M* = 3.74, *SD* = 0.61, *t*(175.07) = 0.17, *p* = .865). In total there were 124 items in the priming task (52 experimental, 52 ambiguous filler, 24 low-ambiguity filler) and 180 items in the test task (78 experimental, 78 ambiguous filler, 24 low-ambiguity filler).

The stimuli that were new to this experiment were recorded by the same speaker as in Experiment 1, and matched with the rest of the stimuli for RMS amplitude. Because the spoken single words were part of a speeded task in this experiment, it was important to more precisely control their onset/offset times than was possible using the sound files from Experiment 1, which included short pre-/post-token silences because of the automated segmentation of these tokens from a continuous recording. For this reason, we manually removed the inaudible periods before and after all of the single word tokens.

#### Procedure

**Figure 4 fig4:**
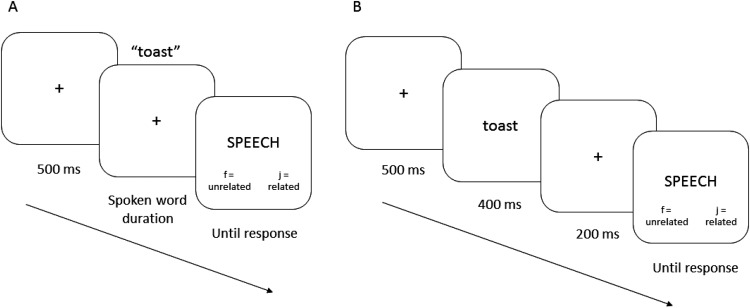
Trial structure for the auditory (A) and visual (B) conditions in the semantic relatedness test task in Experiment 2. In auditory trials, the written probe word was presented on the offset of the spoken target word. In visual trials, the written target word was presented for 400 ms, followed by a 200-ms fixation and the written probe word.

The procedure was the same as that in Experiment 1, apart from the test task. In each trial in the semantic relatedness test, participants were presented with a single words (the target) in either spoken or written form, followed by a written probe word, and made a speeded semantic relatedness judgment (see Figure 4). Trials began with a 500-ms fixation cross in the center of the screen, followed by the auditory or visual target. On auditory trials, the fixation cross remained on the screen while the audio played, and the probe word was presented immediately upon the offset of the sound file. On visual trials, the target was presented in lowercase for 400 ms, followed by a 200-ms fixation (which served as a mask) before the probe word onset. The probe word was always presented visually in upper case, with response key reminders below the word (‘f’ = unrelated, ‘j’ = related). Although it was not possible to perfectly equate the probe presentation times relative to target word recognition in the auditory and visual modalities, we roughly matched the target-probe SOAs by using the mean duration of the auditory sound files (556 ms) as a basis for the 600 ms SOA in the visual condition. In both test modalities, the probe remained on the screen until a valid response was made, and a warning was presented if the response took longer than 1500 ms.[Fig-anchor fig4]

After the test task instructions and examples, participants completed 12 practice trials comprised of 9 low-ambiguity targets and 3 ambiguous filler targets. Half of these target-probe pairs were semantically related (e.g., cheese—BISCUIT) and the other half were unrelated (e.g., soap—DOOR). Half of the practice target words were presented in spoken form and the other half in written form. Feedback on accuracy was presented after each response, and a warning message was shown if the response took longer than 1500 ms. The main task began with 4 filler items, and the remaining items were randomized within the first or second block, with items presented in the same block as in the priming task to minimize between-item variability in prime-to-test durations. There were 3 evenly spaced, optional breaks of up to 1 min during the task.

The task order was: semantic relatedness prime, vocabulary, subjective reading/listening questionnaire, Author Recognition Test (ART), semantic relatedness test. The whole session lasted 42 min on average. The mean estimated delay between the prime and test encounters (i.e., the mean difference between the midpoints of the two tasks) was 23 min.

### Results

Proportions of correct responses in the priming task were near ceiling (*M* = 0.95, *SD* = 0.03, range = 0.83–1.00). Proportions of correct responses on the vocabulary test were in the expected range (*M* = 0.57, *SD* = 0.13, range = 0.26–1.00).

#### Main analysis: Semantic relatedness test

##### Response Times

**Figure 5 fig5:**
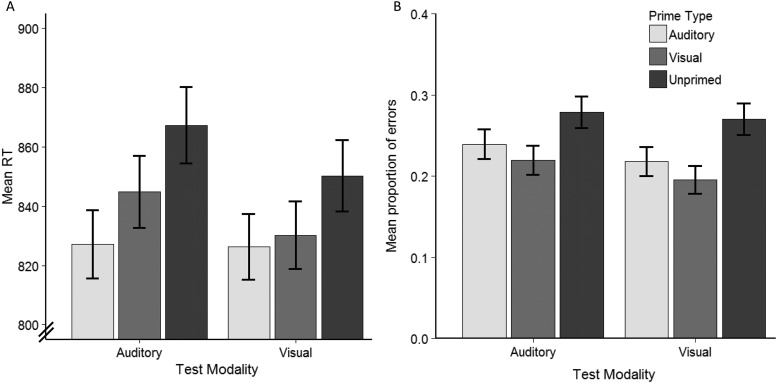
Experiment 2 response times (A; in ms) and proportions of errors (B) in the semantic relatedness test task. Bars are grouped by Test Modality on the *x* axis (Auditory, left bars; Visual, right bars) and color-coded by Prime Type: Auditory (light gray), Visual (medium gray) or Unprimed (dark gray). Bars show the subject grand averages, and error bars show 95% CIs, adjusted to remove between-subjects variance (Morey, 2008).

From the 14118 total responses to experimental items (181 participants × 78 items), there were 9 trials with missing data due to technical problems. Of the remaining responses, any that were incorrect (23.8%) were removed from the RT analysis. This relatively high error rate was expected given the use of subordinate meanings and a speeded task. An examination of accuracy by items revealed that, as expected, the error rates were very variable (range = 0.02–0.69), with higher error rates for words with more subordinate (lower-frequency) meanings (*r*(76) = −0.49, *p* < .001). After plotting accuracy as a function of meaning dominance, there were no target-probe pairs with error rates that deviated greatly from the least squares line, so all items were included. For time-based exclusions of individual trials, we followed the general principle of minimal trimming with model criticism (Baayen & Milin, 2010). We began with liberal cut-off thresholds of less than 300 ms and greater than 2500 ms, then examined the model diagnostic plots for evidence of outliers that would suggest that further trimming is needed. To achieve this, we first determined whether any dependent variable transformation was needed to meet the assumptions of LME models, so that any outliers could be identified after applying the transformation. A comparison of model diagnostic plots (quantile-quantile, distribution of residuals) for the raw, log-transformed and inverse transformed RTs revealed that the log transformation best met the assumptions. The diagnostic plots revealed that no further time-based trimming was necessary. The exclusion of correct responses that were faster than 300 ms (3 trials) or slower than 2,500 ms (21 trials) left 10,732 responses in the analysis. Figure 5A shows the subject grand mean RTs across the 6 conditions. In both test modalities, correct responses to the target-probe pairs were faster for items that were primed than unprimed.[Fig-anchor fig5]

The log-transformed RTs were analyzed with a linear mixed effects model using the same general procedures described in Experiment 1. As in Experiment 1, Prime Type was Helmert-coded (contrast 1: Unprimed = −2/3 vs. Auditory or Visual = 1/3; contrast 2: Auditory = −1/2 vs. Visual = 1/2) and Test Modality was deviation coded (Auditory = −1/2 vs. Visual = 1/2). The model with the full random effects structure did not converge, even after removing the correlations among random effects terms, and after removing the random intercepts. We then removed the term that accounted for the least variance in a stepwise fashion until the model converged, which resulted in the removal of the by-item slope for Test Modality and the by-subject slope for Prime Modality. Thus the final model contained a by-subject random intercept and slopes for Test Modality and the interaction, and a by-item random intercept and slopes for Prime Type and the interaction.

As in Experiment 1, likelihood ratio tests were used to evaluate significance of main effects and interactions. However, unlike logistic mixed effects models, the model summaries for linear mixed effects models created with the lme4 package for R do not return significance tests for fixed effects. To test the significance of the individual model coefficients, we used Satterthwaite’s approximation for degrees of freedom using the ‘lmerTest’ package (Kuznetsova, Brockhoff, & Christensen, 2015), because this method gives acceptable Type I error rates with these models (Luke, 2017).

There was no significant main effect of Test Modality on the RTs, β = −0.01, *SE* = 0.01, *t*(173.73) = −1.76, *p* = .080; χ^2^(1) = 1.91, *p* = .167. The main effect of Prime Type was significant, χ^2^(2) = 30.94, *p* < .001. The model coefficient for the first Prime Type contrast (Unprimed vs. the two primed conditions) was significant, β = −0.04, *SE* = 0.01, *t*(74.64) = −5.69, *p* < .001, and the coefficient for the second Prime Type contrast (Auditory Prime vs. Visual Prime) was marginally significant, β = 0.01, *SE* = 0.01, *t*(61.97) = 1.96, *p* = .055. Pairwise comparisons for Prime Type (with Bonferroni adjustment for multiple comparisons) confirmed that both the Auditory Prime (*z* = −6.11, *p* < .001) and Visual Prime (*z* = −4.19, *p* < .001) conditions produced faster correct semantic relatedness responses than the Unprimed conditions. The Auditory and Visual Prime conditions were not significantly different from one another after correcting for multiple comparisons (*z* = 1.96, *p* = .152).

The model-adjusted mean difference between Auditory Prime and Visual Prime conditions was in the same direction within the two Test Modalities, but numerically larger in the Auditory compared with Visual Test Modality (17 ms and 2 ms, respectively). However, the critical Prime Type × Test Modality interaction was not significant, χ^2^(2) = 3.12, *p* = .210. The model coefficients were not significant for the interaction between Test Modality and the first Prime Type contrast (unprimed vs. primed), β = 0.01, *SE* = 0.01, *t*(102.56) = 0.83, *p* = .409, or between Test Modality and the second Prime Type contrast (Auditory Prime vs. Visual Prime), β = −0.02, *SE* = 0.01, *t*(101.51) = −1.49, *p* = .140.

As in Experiment 1, we compared the relative unstandardized effect sizes for the priming effect versus the critical Prime Modality × Test Modality interaction. The model coefficient and 95% confidence intervals for the priming effect was −0.04 [−0.05, −0.02], whereas for the prime-test modality interaction it was −0.02 [−0.04, 0.01], that is, 50% of the priming coefficient. We also followed up this null finding by computing Bayes Factors to compare the likelihoods of each hypothesis given the data. This analysis revealed that the null hypothesis (no interaction between the prime and test modalities) was about 35 times more likely than the alternative hypothesis, BF_01_ = 35.16.

To determine whether there was significant priming specifically in the two cross-modal conditions, subset analyses were conducted on each cross-modal condition and the unprimed condition with the same test modality (i.e., AV vs. UV, and VA vs. UA). Within each subset, full models were constructed with log RT as the dependent variable, condition (cross-modal primed or unprimed) as a deviance-coded fixed factor, and by-subject and by-item random intercepts and slopes for condition. Likelihood ratio tests showed that the RTs in both cross-modal conditions were significantly faster than RTs in the unprimed conditions with the same test modality (AV vs. UV: β = 0.03, *SE* = 0.01, *t*(48.04) = 3.82, *p* < .001, χ^2^(1) = 12.46, *p* < .001; VA versus UA: β = −0.03, *SE* = 0.01, *t*(55.73) = −3.18, *p* = .002, χ^2^(1) = 9.39, *p* = .002).

##### Accuracy

Of the total 14,109 trials, we excluded any trials with RTs faster than 300 ms (4 trials) and slower than 2500 ms (43 trials), regardless of response accuracy. This was the same criteria used in the RT analysis, and is based on the assumption that these very fast and slow responses reflect accidental button presses and lapses in attention, respectively. The subject grand mean proportions of errors by conditions are shown in Figure 5B. In both test modalities, the proportions of errors were lower when the item was primed versus unprimed, and slightly lower for items primed with a visual versus auditory sentence.

Logistic mixed effects models converged after removing the by-subject slope for Test Modality, thus the final model included a by-subject intercept and slope for Prime Type and the interaction, and a by-item intercept and slope for Prime Type, Test Modality and the interaction. There was a significant main effect of Prime Type, χ^2^(2) = 27.32, *p* < .001. The model coefficients for the first Prime Type contrast (Unprimed vs. the two primed levels) was significant, β = 0.35, *SE* = 0.07, *z* = 5.39, *p* < .001, and the model coefficient for the second Prime Type contrast (Auditory Prime vs. Visual Prime) was also significant, β = 0.21, *SE* = 0.07, *z* = 2.89, *p* = .004. Pairwise comparisons with Bonferroni correction confirmed that all three levels of Prime Type were significantly different from one another; responses were more accurate for items in the Auditory Prime versus Unprimed condition (*z* = 3.47, *p* = .002), for Visual Prime versus Unprimed (*z* = 5.83, *p* < .001) and for Visual Prime versus Auditory Prime (*z* = 2.89, *p* = .012). There was also a significant main effect of Test Modality, β = 0.17, *SE* = 0.06, *z* = 2.68, *p* = .007, χ^2^(1) = 6.77, *p* = .009, with more accurate responses for Visual Test compared with Auditory Test. This was perhaps due to participants occasionally mishearing the spoken target words in the Auditory Test conditions, or to the cost of modality switching between the auditory target and visual probe.

Likelihood ratio tests showed that the Prime Type × Test Modality interaction was not significant, χ^2^(2) = 3.08, *p* = .215. The model coefficient for the interaction between Test Modality and the first Prime Type contrast (primed vs. unprimed) was not significant, β = 0.20, *SE* = 0.11, *z* = 1.76, *p* = .079, nor was the coefficient for the critical interaction with the second Prime Type contrast (Auditory Prime vs. Visual Prime), β = −0.03, *SE* = 0.15, *z* = −0.19, *p* = .849. The model coefficient and 95% confidence intervals for the priming effect was 0.35 [0.23, 0.48], whereas for the prime-test modality interaction it was −0.03 [−0.31, 0.26], that is, ∼9% of the magnitude of the priming coefficient. A Bayesian analysis comparing the full model (alternative) against the model without the 2 × 2 interaction between prime and test modalities (null) showed that, given the data, there was much stronger evidence in favor of the null compared with the alternative hypothesis, *BF*_01_ = 148.41.

We examined the presence of cross-modal priming in the errors using subset analyses for each cross-modal condition (AV and VA) and the unprimed condition with the same test modality (UV and UA, respectively). Within each subset, full models were constructed with accuracy as the dependent variable, condition (cross-modal priming or unprimed) as a fixed factor, and by-subject and by-item random intercepts and slopes for condition. Likelihood ratio tests for the full model against the reduced model (i.e., without condition as a fixed factor) revealed that accuracy was significantly higher in both cross-modal conditions compared with the unprimed conditions with the same test modality (AV vs. UV: β = 0.37, *SE* = 0.09, *z* = 4.01, *p* < .001, χ^2^(1) = 13.81, *p* < .001; VA vs. UV: β = 0.35, *SE* = 0.10, *z* = 3.60, *p* < .001, χ^2^(1) = 10.92, *p* = .001).

#### Participant awareness

After the priming task, none of the participants predicted that the experiment would involve a priming manipulation, and 30 participants mentioned the ambiguous words in the priming sentences. After the test task, 48 participants mentioned the use of ambiguous words, 1 participant’s response indicated awareness of the priming manipulation, and 4 participants mentioned both ambiguity and priming. Using the same categorization criteria as in Experiment 1, there were 68 (38%) ‘Aware’ participants and 113 (62%) ‘Unaware’ participants. Compared with Experiment 1, here there was a greater proportion of participants who noticed the ambiguous words. This increased awareness may have been due to the inclusion of ambiguous filler items in the present experiment, which increased the total number and proportion of ambiguous words in the experiment.

To determine whether the pattern of results was dependent on the ‘aware’ participants, the main analysis was repeated with only the ‘unaware’ participants. For both RT and accuracy analyses, the critical pattern of results was consistent with the main analysis: the ‘unaware’ participants showed significant priming effects and no significant interactions between prime and test modalities (see supplemental materials for detailed results).

### Discussion

Using a speeded semantic relatedness test task, we replicated the main findings of Experiment 1 by showing that cross-modal priming can be observed and that this effect is not significantly reduced compared with unimodal priming. Responses to the ambiguous targets and subordinate-related probe words were significantly faster and more accurate for target words in all primed compared with unprimed conditions. Importantly, the effects of priming were significant in the cross-modal conditions in particular, as revealed by comparisons between each cross-modal condition and the unprimed condition with the same test modality. In the subject mean RTs, there was a numerical interaction characterized by a larger advantage for the auditory prime condition with the auditory compared with visual test. However, this pattern was only partially consistent with the prediction of a unimodal benefit: there was no analogous advantage for the visual prime over auditory prime condition with the visual test. Moreover, there was no such numerical pattern in the error data, and the interaction between prime and test modality was not reliable in either the RT or error analysis. Thus, contrary to our original predictions, and consistent with the results of Experiment 1, we found no evidence for a benefit of modality congruence on word-meaning priming.

There was a significant main effect of test modality in the accuracy analysis in that responses tended to be less accurate in the auditory test conditions. As mentioned in the introduction, we hesitate to draw any conclusions from a main effect of test modality because of the differences in the timing of auditory versus visual target word recognition and relative probe word onset. One possibility is that the (un)certainty of target word recognition differed between modalities, with a greater likelihood of mis-hearing than mis-reading the word. Another possibility is that there was a modality switching cost, making the auditory trials more difficult than the visual trials given that both target word modalities were followed by a visual probe word.

We also found a significant difference between the two prime modalities such that the written prime sentences resulted in slightly but significantly fewer errors at test, as well as marginally slower response times, compared with the spoken prime sentences. Again, there are a number of possible reasons for this difference, including a higher probability of mis-hearing the auditory priming sentences or the ability to spend more time reading the visual sentences, given that the priming task was self-paced. This question could be examined in future research by equating the priming sentence exposure times for auditory and visual conditions with rapid visual serial presentation. Importantly, these main effects do not undermine our ability to draw conclusions about cross-modal priming and the (lack of a) critical prime-test modality interaction.

## General Discussion

The two experiments reported here investigated whether the impact of recent experience on the interpretation of ambiguous words involves predominantly modality-specific or modality-general changes within the lexical-semantic system. We found a clear and consistent pattern of results. There was an overall effect of priming, including significant priming for the cross-modal conditions. Given that previous word-meaning priming studies have used only auditory unimodal conditions, the present work demonstrates for the first time that word-meaning priming occurs in cross-modal and visual unimodal prime-test conditions. Critically, we found no evidence for the hypothesized cross-over interaction between prime and test modality resulting from greater priming in unimodal relative to cross-modal conditions.

In Experiment 1, we examined the effects of modality congruence on word association responses. Word association is the standard test of word-meaning priming and allowed us to verify that priming does occur in unimodal auditory conditions, thus replicating previous results (Betts et al., 2017; Rodd et al., 2016, 2013). In Experiment 2 we sought converging evidence using a speeded semantic relatedness decision task that was chosen to assess the speed and ease of word-meaning access, as opposed to the all-or-nothing measure of meaning preference provided by word association (Cai et al., 2017). Our results with this task were clear in showing significant priming overall and no significant advantage for unimodal over cross-modal conditions in either the response times or accuracy measures. It therefore appears that word-meaning priming affects not only the probability of selecting the primed meaning of ambiguous words, as evidenced by the increase in sentence-consistent word association responses (Experiment 1) and correct semantic relatedness decisions (Experiment 2), but also the speed of access to these meanings, as seen by the faster correct responses in the semantic relatedness test task (Experiment 2).

Compared with short-term priming paradigms, the present studies used relatively high proportions of ambiguous words and primed items. This may have made it more likely that participants noticed the repetition of words across tasks and/or the use of ambiguous words. Indeed, after the test phase, around 1/3 of participants mentioned one or both of these aspects of the study. In Experiment 1, very few participants (3%) mentioned ambiguity after the prime phase. This suggests that awareness of the ambiguity mainly emerged from the test phase, when participants were required to interpret each word in the absence of immediate biasing context. In Experiment 2 the participants who mentioned ambiguity after the prime phase were also in the minority (17%), although this percentage was higher than in Experiment 1, perhaps because the Experiment 2 prime phase contained about twice as many ambiguous items. Importantly, the key results of these experiments were unchanged when we analyzed the subsets of participants who did not express any awareness of the ambiguous words and/or priming manipulation. This is consistent with previous results (Betts et al., 2017; Rodd et al., 2016) and provides further evidence against the possibility that word-meaning priming is attributable to strategic responding in the ‘aware’ participants.

Although the present studies used a Web-based design, the priming effects on word association responses reported here were similar in magnitude to those observed in previous lab-based studies (Rodd et al., 2016, 2013), and our results using a speeded response time measure of word-meaning priming were similarly robust. The recent shift toward conducting experiments online reflects the fact that Internet-based studies provide the ability to collect large amounts of data more quickly and inexpensively, and from a more diverse set of participants, than is generally feasible with lab-based research (Woods, Velasco, Levitan, Wan, & Spence, 2015). This is particularly important given the ongoing issues with low statistical power and false positives in psychological research (Button et al., 2013; Open Science Collaboration, 2015). Although there are a number of important issues to consider when conducting experiments over the Internet (Plant, 2016; Woods et al., 2015), there has been substantial progress made in developing and testing solutions to these issues, with reassuring results (Barnhoorn et al., 2015; Germine et al., 2012; Hilbig, 2016; van Steenbergen & Bocanegra, 2016). In our view, Web-based experiments are a promising avenue for conducting well-powered experimental research with more representative samples.

### The Locus of Word-Meaning Priming

These results are inconsistent with the proposed explanation put forward by Rodd et al. (2013) that word-meaning priming involves changes in form-to-meaning connections. In the Rodd et al. (2004) model and triangle models more generally (Harm & Seidenberg, 2004), form-to-meaning connections determine how orthographic or phonological inputs are mapped onto semantic representations. If word-meaning priming reflects changes to connection weights between form and semantic units then this should produce a stronger bias toward the recently encountered meaning in unimodal than cross-modal conditions. In its strongest form, this account might even predict that cross-modal priming should be absent (because different connection weights mediate access to meaning from written and spoken words). In contrast to this prediction, we found significant priming in all primed conditions and no significant interaction between prime and test modalities.

Null effects are difficult to interpret using frequentist statistics, and do not allow us to make inferences about the absence of an effect of modality congruence on word-meaning priming. However, there are a few reasons why the present results are nonetheless informative. First, we computed Bayes Factors that allowed us to directly compare the likelihoods of the null and alternative hypotheses, given the data. These results showed that the data were more consistent with the absence of an interaction between prime and test modalities (i.e., the null hypothesis), which suggests that our failure to observe a statistically significant interaction was not due to low power. Second, we found that, on average across analyses, the model coefficient for the modality-specific effect was about 1/3 of that for the overall priming effect. This suggests that, even if a true unimodal priming advantage exists, the effect is likely to be small relative to the modality-independent component of word-meaning priming. Finally, the presence of significant cross-modal priming in both experiments is a positive finding that is in clear contradiction with the strongest version of the form-to-meaning hypothesis. Based on these observations, then, we conclude that these results rule out a simple, unimodal form-to-meaning interpretation of the word-meaning priming effect.

Rodd et al. (2016) proposed an alternative to the form-to-meaning hypothesis for the locus of word-meaning priming. They suggested that the changes to the lexical-semantic system could occur within the semantic layer as a result of changes to the recurrent connections between semantic units. These recurrent connections form attractor basins for each separate meaning. During the semantic settling process, there is activation of semantic units from the word form layer which continues until the network reaches a stable semantic representation. As any individual semantic unit becomes activated, the recurrent connections will increase the chances of activation of other units that tended to be coactive during learning. If the prime encounter strengthens these recurrent connections, this would result in an attractor basin that is wider and/or deeper for that particular meaning, making it (a) more likely to be selected, and (b) selected more rapidly (Becker et al., 1997). More generally, this interpretation would suggest that recent experience with word usage causes a long-lasting adjustment to relative meaning dominance levels within the semantic system.

At this point it is unclear *why* the effect of priming would occur through changes to the within-semantic connections, rather than in the form-to-meaning connections. From a communicative point of view, it would make sense for knowledge about word meanings to be modality-general (this is discussed in the later section “Implications for comprehension of written and spoken language”). However, it is not clear whether this effect arises because of a systematic difference in the plasticity of the two types of representations, or because of differences in the representational structures (e.g., sparseness). Meaning representations are relatively sparse and are less likely to overlap across a set words, whereas phonemes/letters are relatively dense and more likely to overlap by chance. Thus any strengthening of form-to-meaning connections as a result of the prime encounter could perhaps be easily cancelled out by subsequent encounters with words that overlap in form but have very different meanings (e.g., bark, bat, break). This explanation could be examined in the future through simulations, and through experiments that manipulate the degree of overlap in form and meaning across words encountered between prime and test.

Another possibility is that the significant cross-modal priming we observed is the result of coactivation of the orthographic and phonological word forms during the prime and/or test phases. Coactivation during the prime phase might result in a strengthening of the form-to-meaning mappings for both phonological and orthographic representations of the word, and would therefore lead to equivalent unimodal and cross-modal priming at test. It is also possible that coactivation of the two word forms occurs during the test encounter, which could result in equivalent unimodal and cross-modal priming, even if the priming encounter produced a modality-specific change to the lexical-semantic network. However, our results are not consistent with previous evidence for a stronger mediating role of phonological than orthographic representations in cross-modal priming (Rueckl & Mathew, 1999), resulting in an asymmetric patterns of cross-modal priming with unspeeded priming sentences and a speeded test task (Monsell, 1985). Nonetheless, at present it is not possible to distinguish between the word form coactivation and modality-general semantic layer explanations.

One concern is that our within-participant manipulations of modality resulted in a bias toward modality-general processing. However, if anything, this design has been shown to produce a bias in the opposite direction, that is, a unimodal over cross-modal priming advantage, compared with a between-subjects manipulation of modality. This finding is thought to reflect voluntary encoding strategies and/or the increased attention drawn to perceptual features of the stimuli in mixed modality trial blocks (Brown, Neblett, Jones, & Mitchell, 1991; Lukatela, Eaton, Moreno, & Turvey, 2007; Mulligan, 2011). This previous work suggests that the present studies may have been biased toward observing a unimodal priming advantage, making the lack of such an advantage even more striking. Whether the use of mixed-modality trials had any biasing effect in word-meaning priming is an open empirical question that could be examined in future work with a between-subjects design and use of a single modality throughout the experiment (Lukatela et al., 2007). Future studies could also investigate the role of dual encoding during priming by presenting visual/auditory masks during the auditory/visual priming sentences to see whether this results in a unimodal priming advantage (Vallet, Brunel, & Versace, 2010).

Our results can also be interpreted within models that propose localist word-meaning nodes. In these models, there are multiple, separate entries for individual word meanings, which compete for selection. Spoken and written word forms map on to the same word-meaning node. We presume that word-meaning priming could be explained in these models in terms of (e.g.) a reduction in the unidirectional inhibitory connection weight from the dominant to the subordinate word-meaning node, so that on the next presentation of the word, the node for the primed (subordinate) meaning will more easily overcome competition from the dominant meaning node and thus be both (a) more likely to be selected and (b) selected more rapidly compared with the unprimed case.

However, we note that localist models have other weaknesses relative to distributed models in their ability to account for lexical-semantic representations. Namely, the representation of a word’s meaning as a single unit, rather than as a more flexible pattern of activation, fails to capture the degrees and ‘shades of meaning’ that occurs in language, and in particular for polysemous words (Rodd et al., 2004). In contrast, distributed connectionist models represent the ‘core’ meaning of polysemous words as a subset of semantic units, and each individual instance or ‘sense’ of the word can be represented as variations on this core semantic pattern. While localist word-meaning nodes are useful for representing words with multiple unrelated meanings because they have no problem implementing one-to-many form-to-meaning mappings, they are thus less parsimonious as an explanation of polysemous word representation since different nodes would be required for even subtle variations to a word’s meaning. They also require that the lexicon makes a categorical distinction between these two types of ambiguity, which is generally considered to be of a more continuous nature ranging from highly related word senses to highly unrelated word meanings but with a large set of intermediate meanings for which classification is unclear. Although these data do not provide any evidence *against* the presence of word nodes, we suggest that they can most parsimoniously be explained in terms of changes in the form-to-meaning connection strengths between *both* auditory and visual word form units and the semantic units, and/or in the shape of the attractor structure within the semantic layer.

Thus far, we have discussed the implications of our results in the context of a single-system account of lexical processing. However, given that learning mechanisms feature heavily in our explanation of the mechanism underlying word meaning priming, it is worth considering alternatives to a single-system account. In particular, a complementary systems account (McClelland, McNaughton, & O’Reilly, 1995) has been highly influential in explaining many aspects of learning and memory processing. In recent years this account has been fruitfully applied to language learning and processing, most particularly in the context of learning the form and meaning of new spoken words (e.g., Dumay & Gaskell, 2007). According to complementary systems models of language, the main repository of lexical knowledge is cortical and relatively stable, whereas new learning is mediated by hippocampal systems to avoid interference with existing knowledge (Davis & Gaskell, 2009). Plausibly, the priming phase of our paradigm resulted in new learning not in the cortical semantic network as described above but in the hippocampus. This new hippocampal representation might act to bind the lexical representation of the ambiguous word with its sentential context, such that the context is able to influence disambiguation of the same word at a later time-point. An involvement of the hippocampus in the comprehension of language on a day-to-day basis has been proposed by Duff and Brown-Schmidt (2012), and helps to explain why hippocampal amnesics show deficits in their ability to retrieve senses of ambiguous words (Klooster & Duff, 2015). We cannot select between single- and multiple-system models on the basis of the current data—future studies of the neural foundations of word-meaning priming or the impact of hippocampal amnesia would be valuable. However, our results do imply that, under a complementary systems account of word-meaning priming, whatever word representations become bound together must be abstract enough to be independent of perceptual modality.

### Implications for Comprehension of Written and Spoken Language

More generally, these results tell us about the degree to which experience with spoken and written language is interlinked. Our data suggest that when listeners/readers update their knowledge about the distributional properties of word meanings in one modality, this experience can impact on how these words are processed in the other modality, and it is possible that this transfer generalizes to other types of linguistic learning and experience. For instance, there is some evidence for transfer across modalities after the consolidation of newly learned words and meanings (van der Ven, Takashima, Segers, & Verhoeven, 2015). There is clear utility in the existence of shared lexical-semantic representations such that comprehension in one modality would generally benefit from knowledge accumulated across all experience with language. Indeed, there is research to suggest that listening comprehension is closely related to reading comprehension in children (e.g., Diakidoy, Stylianou, Karefillidou, & Papageorgiou, 2005; Hagtvet, 2003; Nation & Snowling, 2004) and that reading experience is linked to vocabulary growth across the life span (Sullivan & Brown, 2015). These relationships may be attributable, in part, to knowledge about word usage that is gained through individual encounters with either orthographic or spoken word forms, but which can then be accessed and applied more generally across different types of communication. Although there are additional factors within modalities that have differential effects on comprehension success, such as verbal working memory and orthographic decoding abilities, beyond this it may be that comprehension depends largely on modality-general linguistic knowledge.

However, the modality-general influence of linguistic experience observed here does not preclude the potential for modality-specific learning about word meanings. In particular, if the reader/listener has evidence that words are consistently used to mean different things in different modalities, then we might expect these individuals to keep track of these systematic, modality-specific usage patterns. Evidence that listeners can, in some situations, keep track of how ambiguous words are used differently in specific linguistic environments comes from a recent study of the effect of English accents (British vs. American) on the interpretation of words that have different dominant meanings in the two dialects (e.g., “bonnet”). Cai et al. (2017) found that native British English speakers were more likely to make word association responses related to the American-dominant meaning (e.g., type of hat) than British-dominant meaning (e.g., car part) when the words were spoken in an American accent. However, this shift in preference toward American-dominant meanings did not transfer to written words that were intermixed with the presentation of U.S. accented spoken words which, furthermore, did not differ from written words tested in the absence of any spoken words. Thus, in this case, accented spoken words provide evidence for reliable differences in the likely meanings of words that are used differently by American and British English speakers. Hence, when appropriately signaled, modality-specific interpretations of spoken and written words can be observed. One explanation for these modality-specific effects, in conjunction with the results of the present studies, is that individuals will use their same ‘default’ (dominant) interpretation for both spoken and written ambiguous words, unless there are systematic cues (such as accent) that provide additional information on usage that can bias interpretation toward a different meaning. In the case of the Cai et al. study, the biasing cue (speaker accent) was uniquely present in the spoken words.

This explanation of the differences between the current experiments and Cai et al.’s (2017) results predicts that modality-specific priming might be observed under conditions where participants were exposed to a large number of instances of the ambiguous words, but where the meaning usage differed systematically between the two modalities. It would also predict that individuals could be sensitive to any naturally occurring systematic differences in relative meaning frequencies between spoken and written language. For instance, it might be that slang/colloquial word meanings are used more often in speech than in print (e.g., the British colloquial ‘tired’ meaning of the word “shattered”), and the opposite may be true for more formal word meanings (e.g., the ‘say’ meaning of the word “state”; Purcell-Gates, 2001). In addition, vocabulary in written language has shown to be more diverse than that in spoken language (Chafe & Danielewicz, 1987). Given that most words in English are ambiguous to some extent, the greater formality and variety of vocabulary in written language may have a systematic effect on word meaning frequencies between modalities. Our Experiment 1 results hint at this possibility of different distributional statistics for word meanings in spoken and written language in that the proportion of subordinate meaning word association responses was greater for written than spoken words in the unprimed condition. This might indicate that subordinate meanings are used more often in text, and hence that ambiguous word usage in print is, on average, more balanced in terms of relative meaning frequencies. Unfortunately it is not possible to answer this question with our current data, and because this was an exploratory finding, we note that the difference may be due to chance. Future experiments could examine whether (a) systematic differences exist in the relative frequencies of word meanings in text versus speech, and (b) listeners/readers can keep track of such differences and use them to aid disambiguation.

To summarize, we found that recent exposure to ambiguous words in subordinate-meaning contexts biases later interpretation of those words toward the same meaning, and this biasing effect is not significantly modulated by the (in)congruency between the presentation modalities of the prime and test encounters. Recent experience with ambiguous words therefore appears to influence word meaning interpretation in a modality-general way. These results are not consistent with the previous proposal that word-meaning priming results solely from changes to the connections from either the orthographic or phonological word form to a particular meaning representation. Instead we suggest that the locus of the biasing effect occurs primarily within amodal lexical-semantic representations or as a result of the coactivation of written and spoken word forms, with the result that meanings that were recently encountered become more readily activated and more likely to be retrieved.

## Supplementary Material

10.1037/xlm0000532.supp

## Figures and Tables

**Table 1 tbl1:** Lists of Experimental Ambiguous Words Used in Experiment 1

List 1	List 2	List 3	List 4	List 5	List 6
Bark	Appendix	Ball	Break*	Bow*	Band
Bat	Bar	Bed	Card	Bowl	Cabinet
Bonnet	Box	Bulb	Cricket	Button	Change
Case	Calf	Craft	Cross	Cap	Chest
Cup	Hand	Fan	Deck	Cold	Coach
Joint	Landing	Iron	Gear	Figure	Fence
Lace	Organ	Issue	Jam	Glasses	Key
Pen	Plug	Park	Mark	Gum	Letter
Punch	Spade	Pipe	Mould	Interest^†^	Match
Ring	Stitch	Pupil	Mouse	Nail	Palm
Sink	Straw	Staff	Record*	Note	Sign
Skip	Trailer	Step^†^	Spring	Panel	Speaker
Temple	Wave	Watch	Toast	Trunk	Strike
*Note*. The 78 ambiguous words were pseudorandomly split into 6 lists of 13 words (matched for mean dominance) for the purpose of counterbalancing items across the 6 conditions.					
^†^ Item was included in the analysis of Experiment 1 but removed in Experiment 2. * Item was removed from the word association analyses. Removing these items did not affect the matching of lists for mean dominance.					

**Table 2 tbl2:** Lists of Experimental Ambiguous Words Used in Experiment 2

List 1	List 2	List 3	List 4	List 5	List 6
Bark	Bar	Ball	Calf	Appendix	Band
Bonnet	Figure	Bed	Card	Bank*	Cabinet
Button	Hand	Bulb	Cricket	Bat	Change
Case	Joint	Craft	Cross	Bowl	Chest
Cup	Landing	Iron	Gear	Box	Coach
Fan	Nut*	Issue	Jam	Cap	Fence
Lace	Organ	Park	Log*	Cold	Key
Pen	Plug	Pipe	Mark	Deck	Letter
Punch	Speaker	Pupil	Mould	Glasses	Match
Ring	Spring	Skip	Mouse	Interest	Palm
Sink	Step	Spade	Plant*	Mole*	Sign
Temple	Stitch	Staff	Toast	Nail	Straw
Trailer	Wave	Watch	Trunk	Note	Strike
*Note*. The 78 ambiguous words were pseudorandomly split into 6 lists of 13 words (matched for mean dominance) for the purpose of counterbalancing items across the 6 conditions.					
* Item was new to the stimuli set in Experiment 2.					

**Table 3 tbl3:** List Condition Assignment for Each of the 6 Task Versions in Experiments 1 and 2

List	Version 1	Version 2	Version 3	Version 4	Version 5	Version 6
List 1	AA	AV	VA	VV	UA	UV
List 2	AV	VA	VV	UA	UV	AA
List 3	VA	VV	UA	UV	AA	AV
List 4	VV	UA	UV	AA	AV	VA
List 5	UA	UV	AA	AV	VA	VV
List 6	UV	AA	AV	VA	VV	UA

**Table 4 tbl4:** Experiment 1 Ambiguous Words, Sentences, and Probe Words for the Semantic Relatedness Priming Task

Item	Sentence	Probe	Related
Skip	The home owners were advised to hire a skip	Rubbish	Y
Bat	The fruit bat is a flying mammal	Wings	Y
Sink	There was a soap pump on the side of the sink	Wriggle	N
Case	The crime was suspected to be a case of mistaken identity	Division	N
Bark	The branches and the bark had been damaged by the storm	Closed	N
Lace	The teacher stopped Lily to tie up her lace	Student	Y
Cup	The plaque on the cup was engraved	Win	Y
Bonnet	Petals decorated the brim of the girl’s bonnet	Clothes	Y
Temple	One of the pressure points of the body is the temple	Comb	N
Joint	The police searched the suspected drug dealer and found a joint	Duck	N
Punch	The guests made the most of the free punch	Roast	N
Ring	She picked up the phone to give her daughter a ring	Fry	N
Pen	The pig pen was muddier than ever	Animals	Y
Plug	The plumber had forgotten to put the plug in	Chalk	N
Calf	The muscle in his leg had weakened, particularly the calf	Injury	Y
Appendix	The author put his memos in the appendix of the book.	Writer	Y
Trailer	The new film trailer was released yesterday	Snore	N
Box	The fighter had to box better than he had before	Seat	N
Wave	He couldn’t hear what she said but he saw her wave	Greeting	Y
Straw	The foal was born on the straw in the barn	Horse	Y
Stitch	The athlete was in a lot of pain because of the stitch	Exercise	Y
Organ	The young woman wanted to know how to play the organ	Musician	Y
Landing	There was very little space on the first floor landing	Snail	N
Bar	Access was prevented with a long wooden bar	Slim	N
Spade	The gambler knew that his opponent wanted a spade	Pint	N
Hand	The clock had a broken hand, so it didn’t give the time	Table	N
Park	With his new car, he struggled to park	Ordinary	N
Bulb	The wire connecting the bulb was broken	Wise	N
Fan	Some celebrities don’t interact with members of their fan base	Chief	N
Issue	Alex had edited the most recent issue of the publication	Left	N
Step	She daren’t take one more step in the area	Sugar	N
Racket	The boys tried to watch from their treehouse	Children	Y
Pipe	The grandfather picked up his pipe to smoke	Old	Y
Iron	A simple school science experiment involves iron filings	Students	Y
Craft	There were three funnels along the craft	Color	N
Pupil	Lizzie was the best pupil in the class	Exemplary	Y
Staff	When walking up-hill, the hiker used a staff to help	Mountain	Y
Ball	There were many professional dancers at the ball this year.	Dirt	N
Bed	There were weeds growing in the bed	Garden	Y
Card	She didn’t have a Christmas card to give him	Action	N
Break*	The vase was wrapped so that it wouldn’t break	Protected	Y
Deck	The magician asked the volunteer to pick from the deck	Trick	Y
Mark	The lecturer hadn’t had time to give the essay a mark	Chew	N
Mould	He wanted to use tools to help him mould the statue	Follow	N
Cross	His mum was extremely cross with him	Weather	N
Spring	To make it jump, the toy had a spring inside it	Marry	N
Jam	The roadworks caused a jam all through the town	Construction	Y
Record*	Today was the hottest day on record	Timber	N
Gear	The new employees were told to put on the work gear	Instruction	Y
Mouse	The Apple iMac boasts a new mouse or track-pad device	Technology	Y
Cricket	Only the male cricket can produce a sound	Noise	Y
Toast	The host was asked to make a toast	Speech	Y
Trunk	Pat lifted the lid of the trunk to check what was inside	Look	Y
Nail	She was upset that she had broken her nail	Woman	Y
Figure	All of the bankers knew what the figure would be	Attract	N
Panel	There was a temporary wooden panel separating the two rooms	Wall	Y
Bow*	The girl tied a bow around her ponytail	Daily	N
Note	The lower note suited the singer’s voice better than the higher one	Require	N
Cap	The purchasers were subject to a spending cap	Money	Y
Gum	The boy could feel his new tooth coming through his gum	Fade	N
Interest	The bank charges more interest than others	Face	N
Cold	All the employees had caught the same cold that week	Value	N
Glasses	She poured the champagne into the glasses	Fizz	Y
Bowl	The sportsman’s bowl won the game	Measure	N
Button	Although he was told not to, Fred pushed the button	Disobey	Y
Match	The derby was certainly going to be an exciting match	Rivals	Y
Speaker	Dan connected his iPhone to the speaker	Music	Y
Chest	The large wooden chest was covered in dust	Dirty	Y
Strike	A policy amendment led to a strike across the profession	Union	Y
Coach	Lee was the most respected coach in the business	Add	N
Change	The cashier had given the customer the wrong change	Supermarket	Y
Band	Everyone in the group wore a band	Sky	N
Cabinet	The ministry advised the cabinet on policy alterations	Politicians	Y
Sign	After giving her name and the date, she had to sign the contract	Legal	Y
Letter	The little girl sounded the word out one letter at a time	Stamp	N
Key	The musician had altered the song’s key several times	Behave	N
Palm	The start of the private beach was indicated with a palm	Bite	N
Fence	He wanted to learn how to fence	Sport	Y
* Item was removed from Word Association analysis.			

**Table 5 tbl5:** Experimental and Filler Words, Sentences, and Probe Words Added to the Experiment 2 Semantic Relatedness Priming Task

Item	Sentence	Probe	Related	Type
Bank	The old man had a long way to swim as he headed for the bank.	Horoscope	N	Experimental
Log	They were surprised how well written the old log was.	Berry	N	Experimental
Mole	The woman knew that in one of the embassies there was a mole.	Traitor	Y	Experimental
Nut	The boy wasn’t paying any attention to the bolt when the nut fell off of it.	Require	N	Experimental
Plant	The article reported that it had been very difficult to build the plant.	Industry	Y	Experimental
Ace	The man knew that to win the tennis, it might be enough to get one more ace.	Cash	N	Ambiguous Filler
Article	Dan found that most were broken when looking over all the articles.	Items	Y	Ambiguous Filler
Bail	Joe was pleased that it was not too difficult to pay the bail.	Jail	Y	Ambiguous Filler
Blew	The birthday girl blew out the candles.	Cake	Y	Ambiguous Filler
Bow	The girl tied a bow around her ponytail.	Daily	N	Ambiguous Filler
Branch	Because it was too busy, the young woman couldn’t use that branch.	Soldiers	N	Ambiguous Filler
Break	The vase was wrapped so that it wouldn’t break.	Protected	Y	Ambiguous Filler
Bug	The prisoner was silent because he knew that there were bugs in the room.	Surveillance	Y	Ambiguous Filler
China	She bought a new set of china for the tea party.	Porcelain	Y	Ambiguous Filler
Clip	The woman discovered that the dubbing was very bad in the old clip.	Choice	N	Ambiguous Filler
Coat	The man hoped that it would not take long to paint the new coat.	Layer	Y	Ambiguous Filler
Court	The couple realized that they’d brought the wrong trainers on the way to the court.	Gym	Y	Ambiguous Filler
Crane	The boys by the river watched the injured crane.	Soap	N	Ambiguous Filler
Deed	John wondered whether it had been forged as he thought about the deed.	Ribbon	N	Ambiguous Filler
Dock	The man was kept waiting by the magistrate, even though he was already at the dock.	Prisoner	Y	Ambiguous Filler
Drawer	She kept her gloves and scarves in the drawer.	Town	N	Ambiguous Filler
Drill	The students thought there was too much marching involved when they tried the drill.	Thief	N	Ambiguous Filler
File	The girl recalled that, because it was blunt, the file needed to be replaced.	Antenna	N	Ambiguous Filler
Flour	The woman took the dough and carefully put in the flour she had bought.	Sieve	Y	Ambiguous Filler
Gag	In order to make it funnier the man altered the gag.	Joke	Y	Ambiguous Filler
Grain	The buyer had hoped to find timber with a better quality grain.	Forecast	N	Ambiguous Filler
Gum	The boy could feel his new tooth coming through his gum.	Fade	N	Ambiguous Filler
Hare	The fox tried to chase the hare.	Rabbit	Y	Ambiguous Filler
Isle	There was not room for many harbors on the isle because it was so small.	Video	N	Ambiguous Filler
Knight	The armor was shiny after the knight polished it.	Clean	Y	Ambiguous Filler
Leek	As he cooked, his grandfather joked that this was the biggest ever leek.	Onion	Y	Ambiguous Filler
Lobby	The plans could mean a lot more power for the new lobby.	Campaign	Y	Ambiguous Filler
Male	The researcher thought that it was quite difficult to capture the male.	Gender	Y	Ambiguous Filler
Mint	The man thought that it would be quite easy to run the mint.	Coin	Y	Ambiguous Filler
Model	The designer thought that he had constructed the best ever model.	Tissue	N	Ambiguous Filler
Pair	Since the heel was broken, she bought a new pair of shoes.	Book	N	Ambiguous Filler
Panel	There was a temporary wooden panel separating the two rooms.	Truck	N	Ambiguous Filler
Passage	Bob was relieved that learning the passage had been so easy.	Text	Y	Ambiguous Filler
Poker	Clare knew that, among collectors, this type of poker was very popular.	Politics	N	Ambiguous Filler
Port	The man said that he would like to visit this port.	Ship	Y	Ambiguous Filler
Post	The girl wanted to ask when they would advertise the post.	Employment	Y	Ambiguous Filler
Program	Tom found several viruses when he looked at the program.	Software	Y	Ambiguous Filler
Race	Her reply showed a lack of prejudice when told about her colleague’s race.	Ethnic	Y	Ambiguous Filler
Racket	At lunch time the school children made such a racket.	Elegant	N	Ambiguous Filler
Record	Today was the hottest day on record.	Job	N	Ambiguous Filler
Ruler	The man hoped that there would be a lot more compassion from the new ruler.	Hollywood	N	Ambiguous Filler
Sage	The woman commented that it had not been very easy to consult the sage.	Wise	Y	Ambiguous Filler
Scoop	The woman thought that it would be quite a challenge to write such a big scoop.	Doctor	N	Ambiguous Filler
See	He sat up straight but still couldn’t see.	Warm	N	Ambiguous Filler
Sentence	The old man commented that it was rather harsh after hearing the sentence.	Verdict	Y	Ambiguous Filler
Son	The mother made a packed lunch for her son.	Income	N	Ambiguous Filler
Star	A picture and lots of gossip were included in the article on the star.	Sound	N	Ambiguous Filler
State	The woman described it as very sad, the state her friend lived in.	Camp	N	Ambiguous Filler
Storey	She decided to stop climbing, as she couldn’t manage the third storey.	Building	Y	Ambiguous Filler
Strain	The report said it was a contagious strain he was suffering from.	Germs	Y	Ambiguous Filler
Volume	The man explained why he was not going to publish that volume.	Youth	N	Ambiguous Filler
Whine	Kate complained about the deafening whine in the other room.	Tile	N	Ambiguous Filler
Bread	The loaf of bread was still warm.	Bake	Y	Low-ambiguity filler
Brick	Sally inspected the bricks and noticed some of them were wet.	Gamble	N	Low-ambiguity filler
Cage	An animal cage needs to be cleaned once a week.	Candle	N	Low-ambiguity filler
Desk	When she had finished her coffee, she returned to her desk.	Mug	Y	Low-ambiguity filler
Elm	The blossoms of the elm tree were beautiful.	Shade	Y	Low-ambiguity filler
Fabric	The new fabric felt smooth against his skin.	Bury	N	Low-ambiguity filler
Flag	The flag on the embassy had been vandalized.	Nation	Y	Low-ambiguity filler
Frog	A frog jumped out from underneath the bush.	Scissors	N	Low-ambiguity filler
Guess	Everyone had to guess who the culprit was.	Barrier	N	Low-ambiguity filler
Hill	Alice rolled down the hill in the garden.	Outside	Y	Low-ambiguity filler
Hotel	There weren’t any spare rooms at the hotel.	Vacancy	Y	Low-ambiguity filler
Juice	Orange juice was served in the cafeteria every day.	Food	Y	Low-ambiguity filler
Kitchen	The kitchen had recently been refurbished.	Entertainment	N	Low-ambiguity filler
Lunch	The girls went to lunch together on a daily basis.	Social	Y	Low-ambiguity filler
Meadow	The house has a view of the meadow.	Chisel	N	Low-ambiguity filler
Nest	The boys had discovered an ant’s nest on the patio.	Insect	Y	Low-ambiguity filler
Pond	A fish swam to the edge of the pond.	Fin	Y	Low-ambiguity filler
Request	The employee’s request for better pay was not met.	Education	N	Low-ambiguity filler
Scarf	She chose a scarf to go with her outfit.	Pages	N	Low-ambiguity filler
Snow	An inch of snow had fallen in half an hour.	Boat	N	Low-ambiguity filler
Sugar	The cake was topped with icing sugar.	Agency	N	Low-ambiguity filler
Tractor	The tractor was in need of a good scrub.	Wash	Y	Low-ambiguity filler
Turf	The new turf didn’t survive the frost.	Lawn	Y	Low-ambiguity filler
Vote	People aged eighteen and over were allowed to vote.	Vegetable	N	Low-ambiguity filler

**Table 6 tbl6:** Targets Words, Probe Words, Relatedness, and Item Types Used in the Experiment 2 Semantic Relatedness Test Task

Target	Probe	Related	Item type
appendix	INDEX	Y	Experimental
ball	DANCE	Y	Experimental
band	LOOP	Y	Experimental
bank	RIVER	Y	Experimental
bar	ROD	Y	Experimental
bark	TREE	Y	Experimental
bat	FLY	Y	Experimental
bed	GARDEN	Y	Experimental
bonnet	HAT	Y	Experimental
bowl	THROW	Y	Experimental
box	GLOVE	Y	Experimental
bulb	LAMP	Y	Experimental
button	PRESS	Y	Experimental
cabinet	GOVERNMENT	Y	Experimental
calf	LEG	Y	Experimental
cap	LIMIT	Y	Experimental
card	BIRTHDAY	Y	Experimental
case	TRIAL	Y	Experimental
change	COINS	Y	Experimental
chest	STORAGE	Y	Experimental
coach	TRAINER	Y	Experimental
cold	VIRUS	Y	Experimental
craft	VESSEL	Y	Experimental
cricket	GRASSHOPPER	Y	Experimental
cross	ANGRY	Y	Experimental
cup	TROPHY	Y	Experimental
deck	DEAL	Y	Experimental
fan	SUPPORTER	Y	Experimental
fence	SWORD	Y	Experimental
figure	NUMBER	Y	Experimental
gear	KIT	Y	Experimental
glasses	WINE	Y	Experimental
hand	TIME	Y	Experimental
interest	BANKING	Y	Experimental
iron	STEEL	Y	Experimental
issue	EDITION	Y	Experimental
jam	TRAFFIC	Y	Experimental
joint	DRUG	Y	Experimental
key	TUNE	Y	Experimental
lace	SHOES	Y	Experimental
landing	STAIRS	Y	Experimental
letter	ALPHABET	Y	Experimental
log	JOURNAL	Y	Experimental
mark	GRADE	Y	Experimental
match	GAME	Y	Experimental
mole	SPY	Y	Experimental
mould	SCULPT	Y	Experimental
mouse	KEYBOARD	Y	Experimental
nail	FINGER	Y	Experimental
note	SONG	Y	Experimental
nut	SPANNER	Y	Experimental
organ	MUSIC	Y	Experimental
palm	TROPICAL	Y	Experimental
park	VEHICLE	Y	Experimental
pen	ENCLOSURE	Y	Experimental
pipe	TOBACCO	Y	Experimental
plant	FACTORY	Y	Experimental
plug	DRAIN	Y	Experimental
punch	DRINK	Y	Experimental
pupil	STUDENT	Y	Experimental
ring	PHONE	Y	Experimental
sign	NAME	Y	Experimental
sink	BATHROOM	Y	Experimental
skip	RUBBISH	Y	Experimental
spade	DIAMONDS	Y	Experimental
speaker	EQUIPMENT	Y	Experimental
spring	COIL	Y	Experimental
staff	POLE	Y	Experimental
step	STRIDE	Y	Experimental
stitch	CRAMP	Y	Experimental
straw	HAY	Y	Experimental
strike	UNION	Y	Experimental
temple	FOREHEAD	Y	Experimental
toast	SPEECH	Y	Experimental
trailer	MOVIE	Y	Experimental
trunk	SUITCASE	Y	Experimental
watch	OBSERVE	Y	Experimental
wave	GREET	Y	Experimental
ace	FASHION	N	Ambiguous Filler
article	SERGEANT	N	Ambiguous Filler
bail	OPINION	N	Ambiguous Filler
blew	CALENDAR	N	Ambiguous Filler
bow	EAR	N	Ambiguous Filler
branch	SALT	N	Ambiguous Filler
break	KINGDOM	N	Ambiguous Filler
bug	FAMILY	N	Ambiguous Filler
china	ROAST	N	Ambiguous Filler
clip	UNIVERSITY	N	Ambiguous Filler
coat	WIN	N	Ambiguous Filler
court	BIOLOGY	N	Ambiguous Filler
crane	MAGAZINE	N	Ambiguous Filler
deed	LOUD	N	Ambiguous Filler
dock	STAMP	N	Ambiguous Filler
drawer	FAR	N	Ambiguous Filler
drill	LEAFLET	N	Ambiguous Filler
file	CAPTIVE	N	Ambiguous Filler
flour	THREAD	N	Ambiguous Filler
gag	TORCH	N	Ambiguous Filler
grain	COLONEL	N	Ambiguous Filler
gum	WHIP	N	Ambiguous Filler
hare	BENCH	N	Ambiguous Filler
isle	SPOON	N	Ambiguous Filler
knight	NEWSPAPER	N	Ambiguous Filler
leek	STAGE	N	Ambiguous Filler
lobby	GRIEF	N	Ambiguous Filler
male	SUPPER	N	Ambiguous Filler
mint	OCEAN	N	Ambiguous Filler
model	NORTH	N	Ambiguous Filler
pair	CHALK	N	Ambiguous Filler
panel	KITTEN	N	Ambiguous Filler
passage	TEA	N	Ambiguous Filler
poker	DOCUMENT	N	Ambiguous Filler
port	TOES	N	Ambiguous Filler
post	LEAF	N	Ambiguous Filler
program	FORK	N	Ambiguous Filler
race	BIG	N	Ambiguous Filler
racket	QUEST	N	Ambiguous Filler
record	WEAK	N	Ambiguous Filler
ruler	CLOUD	N	Ambiguous Filler
sage	CELEBRATION	N	Ambiguous Filler
scoop	VAN	N	Ambiguous Filler
see	PLOUGH	N	Ambiguous Filler
sentence	FROGS	N	Ambiguous Filler
son	WHISTLE	N	Ambiguous Filler
star	LEATHER	N	Ambiguous Filler
state	BEER	N	Ambiguous Filler
storey	FEAST	N	Ambiguous Filler
strain	FIRE	N	Ambiguous Filler
volume	CACTUS	N	Ambiguous Filler
whine	CLOTHING	N	Ambiguous Filler
arms	GOAT	N	Ambiguous Filler (Unprimed)
ash	RUDE	N	Ambiguous Filler (Unprimed)
boxer	POTATO	N	Ambiguous Filler (Unprimed)
bridge	CRY	N	Ambiguous Filler (Unprimed)
cell	PENCIL	N	Ambiguous Filler (Unprimed)
cheek	LEASE	N	Ambiguous Filler (Unprimed)
fete	DOG	N	Ambiguous Filler (Unprimed)
flair	ROOM	N	Ambiguous Filler (Unprimed)
fowl	ENGINE	N	Ambiguous Filler (Unprimed)
glare	TEAM	N	Ambiguous Filler (Unprimed)
head	HOLIDAY	N	Ambiguous Filler (Unprimed)
march	PICTURE	N	Ambiguous Filler (Unprimed)
mine	BANANA	N	Ambiguous Filler (Unprimed)
mousse	PRESSURE	N	Ambiguous Filler (Unprimed)
nugget	CARTOON	N	Ambiguous Filler (Unprimed)
ore	CHART	N	Ambiguous Filler (Unprimed)
poll	BEE	N	Ambiguous Filler (Unprimed)
prophet	CHILDREN	N	Ambiguous Filler (Unprimed)
reign	SUPERMARKET	N	Ambiguous Filler (Unprimed)
root	PILL	N	Ambiguous Filler (Unprimed)
shell	TEACHER	N	Ambiguous Filler (Unprimed)
sore	RICE	N	Ambiguous Filler (Unprimed)
stork	NOTEBOOK	N	Ambiguous Filler (Unprimed)
table	HEALTH	N	Ambiguous Filler (Unprimed)
tick	MARRIAGE	N	Ambiguous Filler (Unprimed)
wait	FRIEND	N	Ambiguous Filler (Unprimed)
bread	WHEAT	Y	Low-Ambiguity Filler
brick	CEMENT	Y	Low-Ambiguity Filler
cage	SHADY	N	Low-Ambiguity Filler
desk	WORK	Y	Low-Ambiguity Filler
elm	STRING	N	Low-Ambiguity Filler
fabric	SOFT	Y	Low-Ambiguity Filler
flag	COUNTRY	Y	Low-Ambiguity Filler
frog	HOP	Y	Low-Ambiguity Filler
guess	NECK	N	Low-Ambiguity Filler
hill	CHIN	N	Low-Ambiguity Filler
hotel	SLEEP	Y	Low-Ambiguity Filler
juice	WAR	N	Low-Ambiguity Filler
kitchen	STOVE	Y	Low-Ambiguity Filler
lunch	MEAL	Y	Low-Ambiguity Filler
meadow	GRASS	Y	Low-Ambiguity Filler
nest	WRITE	N	Low-Ambiguity Filler
pond	WATER	Y	Low-Ambiguity Filler
request	ANIMAL	N	Low-Ambiguity Filler
scarf	REST	N	Low-Ambiguity Filler
snow	MAGNET	N	Low-Ambiguity Filler
sugar	WRIST	N	Low-Ambiguity Filler
tractor	STAPLE	N	Low-Ambiguity Filler
turf	BRIGHT	N	Low-Ambiguity Filler
vote	BALLOT	Y	Low-Ambiguity Filler

## Footnotes

1 An anonymous reviewer suggested that we use the participants’ self-coded responses, because of the possibility that our interpretation of their response was incorrect. For instance, a participant could respond to the target word BANK with ‘river’ because their local financial institution (dominant meaning) is located near a river, not because they interpreted the target word with the ‘edge of a river’ (subordinate) meaning. Therefore we also analyzed the responses using the participants’ original self-coding, and the pattern of the results was the same: there were no changes in which effects reached the criterion for significance.

2 The unprimed data show that 80% of the word meanings used in the priming task were (as intended) subordinate (proportions of consistent responses less than 0.4), 16% were balanced (0.4-0.6), and 4% of the meanings were dominant in our sample (above 0.6).

3 During this model building procedure, by-subject intercept was removed, but was added back in after the model failed to converge with the last remaining by-subject random effect term.

4 The BIC approximation method for Bayes Factors has the advantage of being a straightforward solution for mixed effects models, however we note that BICs have been criticized for biasing toward the simpler model, that is, the null hypothesis (Vandekerckhove, Matzke, & Wagenmakers, 2014; Weakliem, 1999).

## Experimental Stimuli and Stimuli-Condition Assignment

[Table-anchor tbl1][Table-anchor tbl2][Table-anchor tbl3][Table-anchor tbl4][Table-anchor tbl5][Table-anchor tbl6]
