# Supplementary material for: Listeners and Readers Generalize Their Experience With Word Meanings Across Modalities
Source: J Exp Psychol Learn Mem Cogn. 2018 Feb 1;44(10):1533–61. doi: 10.1037/xlm0000532 (PMC6179175; doi:10.1037/xlm0000532)
Supplement: Supplementary file 1 [file modality_supplemental_material_final.docx]

# Supplemental Material for Gilbert, Davis, Gaskell & Rodd

## Analysis of unaware participant subsets

The main analyses were repeated on the subset of participants who did not report any awareness of either the priming manipulation or the use of ambiguous words. In all of these subset analyses, the models used the same random effects structure as that in the main analysis.

### Experiment 1

The results were consistent with those reported in the main analysis, with the exception that effect of Test Modality was significant in the main analysis and non-significant in the subset of ‘unaware’ participants, *β* = 0.13, SE = 0.08, *z* = 1.59, *p* = .111; *χ*^2^(1) = 2.50, *p* = .114. The main effect of Prime Type was significant, *χ*^2^(2) = 32.83, *p* < .001. There was a significant effect of priming, *β* = 0.48, SE = 0.09, *z* = 5.64, *p* < .001, and no significant difference between Auditory and Visual Prime conditions, *β* = 0.07, SE = 0.09, *z* = 0.77, *p* = .443. Bonferroni-corrected pairwise comparisons showed that there were significantly more subordinate meaning responses in both the Auditory Prime (*z* = 4.59, *p* < .001) and Visual Prime (*z* = 5.32, *p* < .001) conditions compared to the Unprimed conditions, and that the Auditory Prime and Visual Prime conditions were not significantly different (*z* = 0.77, *p* = .441).

There was no significant interaction between Prime Type and Test Modality, *χ*^2^(2) = 0.77, *p* = .680. The interaction between Test Modality and the first Prime Type contrast, primed vs unprimed, was non-significant, *β* = -0.13, SE = 0.17, *z* = -0.73, *p* = .465. More importantly, the model coefficient for the interaction between Test Modality and the second Prime Type contrast, Auditory Prime vs Visual Prime, was also non-significant, *β* = 0.09, SE = 0.19, *z* = 0.50, *p* = .619.

### Experiment 2

*Response times.* The pattern of results was generally consistent with the main analysis. The only difference was that in the main analysis there was no main effect of Test Modality, whereas this effect was significant in the ‘unaware’ group of participants, *β* = -0.02, SE = 0.01, *t*(112) = -2.26, *p* = .026; *χ*^2^(1) = 5.06, *p* = .025. The effect was due to faster responses for Visual Test vs Auditory Test conditions in this group.

The main effect of Prime Type was significant, *χ*^2^(2) = 20.47, *p* < .001. Correct responses were faster for items in the primed vs unprimed conditions, *β* = -0.03, SE = 0.01, *t*(6133) = -4.31, *p* < .001. There was no significant difference between the Auditory Prime and Visual Prime conditions, *β* = 0.01, SE = 0.01, *t*(6153) = 1.46, *p* = .146. Pairwise comparisons with Bonferroni correction showed that responses to Unprimed items were slower than to items in both the Auditory Prime (*z* = -4.45, *p* < .001) and Visual Prime (*z* = -3.05, *p* = .007) conditions, and that the Auditory and Visual Prime conditions did not significantly differ (*z* = 1.46, *p* = .436).

There was no significant interaction between Prime Type and Test Modality, *χ*^2^(2) = 1.37, *p* = .505. The model coefficients was not significant for the interaction between Test Modality and the first Prime Type contrast (unprimed vs primed), *β* = 0.02, SE = 0.02, *t*(113) = 0.98, *p* = .321. Importantly, the interaction between Test Modality and the second Prime Type contrast (Auditory Prime vs Visual Prime) was remained non-significant in this subset, *β* = -0.01, SE = 0.02, *t*(75) = -0.57, *p* = .570.

*Accuracy.* The results in the ‘unaware’ group followed the same pattern as in the main analysis. There was a significant effect of Test Modality, with more correct responses to the Visual Test than Auditory Test conditions, *β* = 0.16, SE = 0.07, *z* = 2.34, *p* = .019; *χ*^2^(1) = 5.23, *p* = .022. There was also a significant effect of Prime Type on response accuracy, *χ*^2^(2) = 22.02, *p* < .001. There were more correct responses to primed vs unprimed items, *β* = 0.33, SE = 0.08, *z* = 4.47, *p* < .001, and to items in the Visual Prime vs Auditory Prime conditions, *β* = 0.26, SE = 0.09, *z* = 3.06, *p* = .002. Bonferroni-corrected pairwise comparisons showed that both the Auditory Prime and Visual Prime conditions produced significantly more correct responses than the Unprimed condition (*z* = 2.53, *p* = .035, and *z* = 3.06, *p* = .007, respectively).

There was no significant interaction between Prime Type and Test Modality, *χ*^2^(2) = 2.73, *p* = .256. The model coefficient for the interaction between Test Modality and the first Prime Type contrast (unprimed vs primed) was not significant, *β* = 0.21, SE = 0.14, *z* = 1.50, *p* = .135. The coefficient for the critical interaction between Test Modality and the second Prime Type contrast (Auditory vs Visual) was also non-significant, *β* = 0.15, SE = 0.16, *z* = 0.89, *p* = .376.

## Experiment 2 range analysis

In addition to the Experiment 2 main analyses, the use of the speeded test provided the opportunity to conduct exploratory analyses to investigate the time-course of priming effects using a range analysis (Lindsay, Sedin, & Gaskell, 2012; McQueen, 1991; Pitt & Samuel, 1993). Specifically we were interested in whether the effect of priming can be observed across the whole distribution of RTs, or whether it is driven primarily by differences in the slower responses (i.e. the right-hand tails of the distributions). The latter would suggest that priming effects emerged relatively late in the semantic decision process, with individuals being more likely to make very slow responses to unprimed targets, and more incorrect responses on these slow trials. By contrast, effects of priming in both the fast and slow ranges would indicate that priming effects can also emerge earlier in the decision process. Here we only compared primed and unprimed conditions because the main analyses revealed no reliable effects of modality on priming.

Using a strategy similar to that used by Lindsay et al. (2012), we categorised responses as being either fast or slow, while taking into account variability due to the participant, item, and condition associated with each individual data point. An intercepts-only LME model was run on the log-transformed RTs, and for each trial, the relevant item intercept that was estimated by the model was subtracted from the log-transformed RT. These adjusted response times were then categorised as fast or slow, where trials with adjusted log RT values less than the participant/condition median were considered ‘fast’ and those above this median were ‘slow’. When there was an odd number of data points, the middle value was treated as ‘slow’^[[1]](#footnote-1)^. This resulted in similar numbers of (relatively) fast and slow responses across conditions^[[2]](#footnote-2)^. This categorisation procedure was done separately for the RT analysis (only correct responses) and the accuracy analysis (both correct and incorrect responses). Because of the difference in these data sets, the categorisation of the correct responses as either fast or slow differed slightly between the two analyses^[[3]](#footnote-3)^.

Figure 1 shows the subject grand means for RTs and proportions of errors by priming condition and speed category. Overall, there were more errors in the fast than slow category, reflecting the speed-accuracy trade-off that is characteristic of speeded decision making tasks. For the RT analyses, LME models were run on the log-transformed RTs with priming condition (primed or unprimed) as a fixed factor, and with by-item and by-subject random intercepts and slopes for priming condition. Significance tests for model coefficients (with Satterthwaite’s approximation for degrees of freedom) and likelihood ratio tests comparing the full model to a model without a fixed effect for priming condition revealed a significant effect of priming in both the relatively fast responses, *β* = -0.03, SE = 0.01, *t*(176.1) = -5.42, *p* < .001, *χ*^2^(1) = 25.89, *p* < .001, and slow responses, *β* = -0.03, SE = 0.01, *t*(87.7) = -4.62, *p* < .001, *χ*^2^(1) = 18.91, *p* < .001. In both subsets, the correct responses were faster when the target word was primed than unprimed. We ran the same set of analyses on the accuracy data and found a significant effect of priming in both the fast responses, *β* = 0.42, SE = 0.09, *z* = 4.57, *p* < .001, *χ*^2^(1) = 15.67, *p* < .001, and in the slow responses, *β* = 0.24, SE = 0.08, *z* = 2.94, *p* = .003, *χ*^2^(1) = 7.20, *p* = .007. In both subsets, there were more correct responses for primed items compared to unprimed items. Together these RT and accuracy results show that, rather than being limited to the relatively slow semantic relatedness decisions, priming effects are evident across the whole range of response times.

Figure 1. Experiment 2 response times (A; in ms) and proportions of errors (B) in the semantic relatedness test task. Bars are grouped by response speed category on the x-axis (Fast, left bars; Slow, right bars; see text for categorisation details) and colour-coded by priming condition (Primed, light gray; Unprimed, dark gray). Bars show the subject grand averages, and error bars show 95% CIs, adjusted to remove between-subject variance (Morey, 2008).

Because we used a speeded task to examine access to word meanings, we were able to examine the time-course of priming effects. Separate analyses of the fast and slow responses (categorised relative to the item and participant distributions, and within priming conditions) revealed significant priming effects in both speed categories. This result shows for the first time that word-meaning priming is not entirely due to differences emerging at later stages of the decision making process, but instead appears to affect word interpretation and meaning access even in earlier stages.

1. The analyses were also run with the middle data points categorised as ‘fast’, and the pattern of results did not change for either the RT or accuracy analyses. [↑](#footnote-ref-1)
2. In the RT analysis, there were 5278 fast and 5454 slow responses, and an average of 29.7 trials per participant in each speed category. In the accuracy analysis, there were 7010 fast responses and 7052 slow responses, and an average of 38.8 trials per participant in each speed category. [↑](#footnote-ref-2)
3. From the RT to accuracy categorisation, 4.8% of correct responses changed from ‘fast’ to ‘slow’ and 2.1% changed from ‘slow’ to ‘fast’. [↑](#footnote-ref-3)
